# Correcting for bias due to mismeasured exposure history in longitudinal studies with continuous outcomes

**DOI:** 10.1111/biom.13877

**Published:** 2023-05-24

**Authors:** Jiachen Cai, Ning Zhang, Xin Zhou, Donna Spiegelman, Molin Wang

**Affiliations:** 1 Department of Biostatistics, Yale School of Public Health, New Haven, Connecticut, USA; 2 Department of Epidemiology, Harvard T.H. Chan School of Public Health, Boston, Massachusetts, USA; 3 Center for Methods in Implementation and Prevention Science, Yale School of Public Health, New Haven, Connecticut, USA; 4 Department of Biostatistics, Harvard T.H. Chan School of Public Health, Boston, Massachusetts, USA; 5 Channing Division of Network Medicine, Harvard Medical School, Brigham and Women’s Hospital, Boston, Massachusetts, USA

**Keywords:** air pollution, cognitive decline, generalized estimating equation, longitudinal data, measurement error, PM_2.5_

## Abstract

Epidemiologists are often interested in estimating the effect of functions of time-varying exposure histories in relation to continuous outcomes, for example, cognitive function. However, the individual exposure measurements that constitute the history upon which an exposure history function is constructed are usually mismeasured. To obtain unbiased estimates of the effects for mismeasured functions in longitudinal studies, a method incorporating main and validation studies was developed. Simulation studies under several realistic assumptions were conducted to assess its performance compared to standard analysis, and we found that the proposed method has good performance in terms of finite sample bias reduction and nominal confidence interval coverage. We applied it to a study of long-term exposure to PM_2.5_, in relation to cognitive decline in the Nurses’ Health Study Previously, it was found that the 2-year decline in the standard measure of cognition was 0.018 (95% CI, −0.034 to −0.001) units worse per 10 *μ*g/m^3^ increase in PM_2.5_ exposure. After correction, the estimated impact of PM_2.5_ on cognitive decline increased to 0.027 (95% CI, −0.059 to 0.005) units lower per 10 *μ*g/m^3^ increase. To put this into perspective, effects of this magnitude are about 2/3 of those found in our data associated with each additional year of aging: 0.044 (95% CI, −0.047 to −0.040) units per 1 year older after applying our correction method.

## 1 | INTRODUCTION

Longitudinal continuous health endpoints are common in epidemiological research, through data gathered on repeated continuous health endpoints obtained from multiple follow-up questionnaires. Changes in trajectories of body mass index, cognitive function, CD4 cell counts, and viral loads are examples of these objects of study. The exposure of interest, which is often a function of the exposure history, is typically mismeasured due to the widespread presence of measurement error in individual exposure measurements, thus leading to biased estimates of exposure effects on the health outcome (Gerharz et al., 2013).

This research is motivated by a study of PM_2.5_ in relation to cognitive decline, in the Cognitive Cohort of the Nurses’ Health Study (NHS), a long-term prospective cohort study of U.S. female registered nurses. NHS began in 1976, collecting updated information through mail-based questionnaires that include questions on lifestyle and new diagnoses of health outcomes every 2 years (Colditz et al., 1997). At the inception of the study, there were 121,700 participants, aged 30–55 years old. The NHS Cognitive Cohort, which began in 1995, comprised 19,409 U.S. women over age 70. In a previous study about this cohort, it was found that long-term exposure to PM_2.5_ (particulate matter ≤2.5 *μ*m) was associated with significantly worse cognitive decline; every 10 *μ*g/m^3^ greater exposure to PM_2.5_ was cognitively equivalent to aging by approximately 2 years (Weuve et al., 2012). The effect of chronic PM_2.5_ exposure on cognitive decline was likely underestimated because the exposure data were a mismeasured monthly exposure measurement, constructed from a spatiotemporal model based on the nearest monitor of a person’s residential address. These data have been found to be measured with substantive error compared with the gold standard, average monthly personal exposure, with a correlation coefficient equal to 0.35 (Kioumourtzoglou et al., 2014). To validly and efficiently assess the effect of environmental exposures on changes in health outcomes across the life course, exposure measurement error needs to be taken into account in the analysis.

Although statistical methods have been proposed for analyzing longitudinal data in the presence of measurement error, existing methods have substantial limitations that need to be addressed to be useful for longitudinal environmental health studies. First, most methods apply only to a simple updated exposure variable following the classical additive measurement error model (MEM) for all subjects at all time points. Existing methods do not apply to mismeasured exposure variables that do not follow this simple measurement error structure, nor to exposure variables that are functions of a mismeasured exposure history (Battauz et al., 2011; Dumitrescu, 2010; Liang, 2009; Xiao et al., 2010a, 2010b). Second, many existing methods are not suitable for the main study (MS)/validation study (VS) design (Zhang et al., 2018), which is common in environmental health studies. Although methods able to address the above concerns exist for survival data endpoints such as mortality (Liao et al., 2018), as far as we know, there are no methods available for solving these limitations when the health endpoints are continuous and longitudinal.

This paper develops a novel method to solve this long-standing issue, targeting the case where the exposure is mismeasured while assuming the outcome is correctly measured. Although motivated by a study of cognitive decline, the method is fully general and will be applicable to any longitudinal study with repeated measures in continuous outcomes and exposure variables that are functions of the mismeasured exposure history. The remainder of this paper is organized as follows: Section 2 elucidates the proposed method for correcting mismeasured exposures, Section 3 assesses the finite sample properties of the new approach under realistic scenarios expected to be encountered in practice by simulation studies, and in Section 4, our proposed method is applied to analyze the motivating environmental health example. We close in Section 5 with conclusions and discussion.

## 2 | METHODS

### 2.1 | Notation and models

Consider a longitudinal MS/VS design, where the sample sizes in the MS and VS are $n_{1}$ and $n_{2}$, respectively. Let Y(t),C(t), and W(t) represent observed outcome, mismeasured exposure (also called surrogate exposure in this work), and a vector of potential confounders at time point t, respectively. For each individual $i\left(i=1,2,\ldots ,n_{1}\right)$ in the MS, the observed data include $m_{i}$ measurements $\left(Y_{i}(t_{i1}),\ldots ,Y_{i}(t_{im_{i}}),C_{i}(t_{i1}),\ldots ,C_{i}(t_{im_{i}}),W_{i}(t_{i1}),\ldots ,W_{i}(t_{im_{i}})\right)$ at time $t_{ij},j=1,2,\ldots ,m_{i}$. In the VS, $\left(c_{i}(t_{i1}),\ldots ,c_{i}(t_{{il}_{i}})\right)$ denotes $l_{i}$ measurements of true exposure at time $t_{ij}$ for the *i*th individual, where $j=1,\ldots ,l_{i}$ and $i=n_{1}+1,\ldots ,n_{1}+n_{2}$. In the main manuscript below, we provide derivations for VS with a single measurement $\left(l_{i}=1\right.$); formulas for the VS with repeated measurements $\left(l_{i}>1\right)$ are also provided in Web Appendix A.2. There are two types of VS designs: internal validation study (IVS) and external validation study (EVS). In the IVS, the VS is a subsample of the MS. Thus, outcome status is available and the observed data for participant i in the IVS are $\left(Y_{i}(t_{i1}),C_{i}(t_{i1}),W_{i}(t_{i1}),t_{i1},c_{i}(t_{i1})\right)$. However, in the EVS, where participants are often from a different group, the outcome variable is not available; so the observed data for participant i in the VS are $\left(C_{i}\left(t_{i1}\right),W_{i}\left(t_{i1}\right),t_{i1},c_{i}\left(t_{i1}\right)\right)$. We assume transportability between the MS and IVS or EVS. That is, the parameters of the MEM in the VS that generate the validation data are the same as those in the MS. In an MS/IVS, this assumption can and should be empirically verified through standard statistical methods comparing the two model fits and the estimates from the two data sources.

Let $\tilde{C}(t),\tilde{c}(t)$, and $\tilde{W}(t)$ denote the history of surrogate exposure, true exposure, and potential confounders, up to time point t, respectively: ${\tilde{C}}_{i}(t_{ij})=\left\{C_{i}(t_{i1}),\ldots ,C_{i}(t_{ij})\right\},{\tilde{c}}_{i}(t_{ij})=\left\{c_{i}(t_{i1}),\ldots ,c_{i}(t_{ij})\right\}$ and ${\tilde{W}}_{i}(t_{ij})=\left\{W_{i}(t_{i1}),\ldots ,W_{i}(t_{ij})\right\}$. In addition, let ${\tilde{t}}_{ij}=\left\{t_{i1},\ldots ,t_{ij}\right\}$. Let g(.) denote the function of the exposure history that is of interest. For example, in a particular time window, a moving average exposure is defined as the average measurement, and a cumulative exposure is defined as the total exposure (Wang et al., 2016). Specifically, a b-month moving average can be quantified as $\frac{\int_{t-b}^{t}c(s)ds}{b}$, which be approximated by $\frac{\sum_{s=t-b}^{t}I(s)c(s)}{\sum_{s=t-b}^{t}I(s)}$, where I(s) is a missing indicator, taking the value of 1 if c(s) is available and 0 otherwise. Similarly, a b-month cumulative exposure can be calculated as $\int_{t-b}^{t}c(s)ds$, which can be approximated by $\sum_{s=t-b}^{t}I(s)c(s)$. Generally, the exposure history function based on the true exposure, $\tilde{c}(t)$, is $X_{i}(t_{ij})=g(\tilde{c_{i}}(t_{ij}))=g(c_{i}(t_{i1}),\ldots c_{i}(t_{ij}))$ and its mismeasured counterpart is $Z_{i}(t_{ij})=g({\tilde{C}}_{i}(t_{ij}))=g(C_{i}(t_{i1}),\ldots C_{i}(t_{ij}))$.

To elucidate the basic methodologic strategy, we assume the following linearly divergent difference (LDD) model (Frison & Pocock, 1997) with a scalar exposure variable

(1) $$𝔼\left[Y_{i}\left(t_{ij}\right)|{\tilde{c}}_{i}\left(t_{ij}\right),{\tilde{t}}_{ij},{\tilde{W}}_{i}\left(t_{ij}\right)\right]=\beta_{0}+\beta_{1}X_{i}\left(t_{ij}\right)+\beta_{2}t_{ij}\;+\beta_{3}X_{i}\left(t_{ij}\right)t_{ij}+\beta_{4}^{T}W_{i}\left(t_{ij}\right),$$

where superscript T represents transpose, and unless otherwise noted, all vectors are column vectors throughout this paper. We henceforth refer to this model as the outcome model. In this model, the parameter of interest is $\beta_{3}$, as it measures the extent to which the trajectory of the outcome variable over time changes as a result of the exposure.

In the MEM, we use function f(⋅) to denote the relationship between the true and surrogate exposures; that is,

(2) $$𝔼\left[c_{i}\left(t_{ik}\right)|C_{i}\left(t_{ik}\right),t_{ik},W_{i}\left(t_{ik}\right)\right]=f\left(C_{i}\left(t_{ik}\right),t_{ik},W_{i}\left(t_{ik}\right);\alpha \right),$$

where α denotes the unknown regression coefficients in the MEM.

### 2.2 | The method

To obtain the induced model in the MS taking exposure measurement error into account, we apply the formula of iterated expectation:

$$𝔼\left[Y_{i}\left(t_{ij}\right)|{\tilde{C}}_{i}\left(t_{ij}\right),{\tilde{t}}_{ij},{\tilde{W}}_{i}\left(t_{ij}\right)\right]=𝔼[𝔼[Y_{i}\left(t_{ij}\right)|{\tilde{c}}_{i}\left(t_{ij}\right),\;{\tilde{C}}_{i}\left(t_{ij}\right),{\tilde{t}}_{ij},{\tilde{W}}_{i}\left(t_{ij}\right)]|{\tilde{C}}_{i}\left(t_{ij}\right),{\tilde{t}}_{ij},{\tilde{W}}_{i}\left(t_{ij}\right)].$$

Assuming that $𝔼\left[Y_{i}(t_{ij})|\tilde{c_{i}}(t_{ij}),{\tilde{C}}_{i}(t_{ij}),{\tilde{t}}_{ij},{\tilde{W}}_{i}(t_{ij})\right]=𝔼\left[Y_{i}(t_{ij})|\tilde{c_{i}}(t_{ij}),{\tilde{t}}_{ij},{\tilde{W}}_{i}(t_{ij})\right]$ (the surrogacy assumption), the above equation can be further written as:

(3) $$\;𝔼\left[Y_{i}\left(t_{ij}\right)|{\tilde{C}}_{i}\left(t_{ij}\right),{\tilde{t}}_{ij},{\tilde{W}}_{i}\left(t_{ij}\right)\right]\;\;=𝔼[\beta_{0}+\beta_{1}X_{i}\left(t_{ij}\right)+\beta_{2}t_{ij}\;\;+\beta_{3}X_{i}\left(t_{ij}\right)t_{ij}+\beta_{4}^{T}W_{i}\left(t_{ij}\right)|{\tilde{C}}_{i}\left(t_{ij}\right),{\tilde{t}}_{ij},{\tilde{W}}_{i}\left(t_{ij}\right)]\;=\beta_{0}+\beta_{1}𝔼\left[X_{i}\left(t_{ij}\right)|{\tilde{C}}_{i}\left(t_{ij}\right),{\tilde{t}}_{ij},{\tilde{W}}_{i}\left(t_{ij}\right)\right]\;\;+\beta_{2}t_{ij}+\beta_{3}t_{ij}𝔼\left[X_{i}\left(t_{ij}\right)|{\tilde{C}}_{i}\left(t_{ij}\right),{\tilde{t}}_{ij},{\tilde{W}}_{i}\left(t_{ij}\right)\right]\;\;+\beta_{4}^{T}W_{i}\left(t_{ij}\right).$$

It remains to derive $𝔼\left[X_{i}(t_{ij})|{\tilde{C}}_{i}(t_{ij}),{\tilde{t}}_{ij},{\tilde{W}}_{i}(t_{ij})\right]$, which represents the relationship between the true and surrogate exposure history. The above development is general, and this approach is applicable to different exposure history functions. Here, we illustrate the approach with the cumulative average exposure, as it is the exposure metric of interest in our illustrative example. Then, $X_{i}(t_{ij})=\frac{\sum_{k=1}^{j-1}c_{i}(t_{ik})(t_{i(k+1)}-t_{ik})}{t_{ij}-t_{i1}}$,

(4) $$𝔼\left[X_{i}(t_{ij})|{\tilde{C}}_{i}(t_{ij}),{\tilde{t}}_{ij},{\tilde{W}}_{i}(t_{ij})\right]=\frac{\sum_{k=1}^{j-1}(t_{i(k+1)}-t_{ik})E\left[c_{i}(t_{ik})|C_{i}(t_{i1}),\ldots ,C_{i}(t_{ij}),t_{i1},\ldots ,t_{ij},W_{i}(t_{i1}),\ldots ,W_{i}(t_{ij})\right]}{t_{ij}-t_{i1}}.$$

We further make the localized error assumption (Zucker & Spiegelman, 2008); that is,

$$𝔼\left[c_{i}(t_{ik})|C_{i}(t_{i1}),\ldots ,C_{i}(t_{ij}),t_{i1},\ldots ,t_{ij},W_{i}(t_{i1}),\ldots ,W_{i}(t_{ij})\right]\;\;=𝔼\left[c_{i}(t_{ik})|C_{i}(t_{ik}),t_{ik},W_{i}(t_{ik})\right]\;\;=f(C_{i}(t_{ik}),t_{ik},W_{i}(t_{ik});\alpha ).$$

Note that this assumption is empirically verifiable in validation studies with repeated measurements on the same participants, otherwise not (see more details in Section 5).

Then, it follows that

(5) $$𝔼\left[X_{i}\left(t_{ij}\right)|{\tilde{C}}_{i}\left(t_{ij}\right),{\tilde{t}}_{ij},{\tilde{W}}_{i}\left(t_{ij}\right)\right]\;=\frac{\sum_{k=1}^{j-1}\left(t_{i(k+1)}-t_{ik}\right)f\left(C_{i}\left(t_{ik}\right),t_{ik},W_{i}\left(t_{ik}\right);\alpha \right)}{t_{ij}-t_{i1}},\;j=2,3,\ldots ,m_{i}.$$

Although we assume a linear model

(6) $$f\left(C_{i}\left(t_{ik}\right),t_{ik},W_{i}\left(t_{ik}\right);\alpha \right)=\alpha_{0}+\alpha_{1}C_{i}\left(t_{ik}\right)+\alpha_{2}t_{ik}\;\;+\alpha_{3}C_{i}\left(t_{ik}\right)t_{ik}+\alpha_{4}^{T}W_{i}\left(t_{ik}\right)$$

for the MEM in the following simulation study and data application, it is easy to show that our method can accommodate a nonlinear association in MEM as well.

### 2.3 | Estimation

Let α and β be the column vectors of unknown parameters in the measurement error and outcome models, respectively; thus, the full set of parameters to be estimated is $\theta ={\left[\alpha^{T},\beta^{T}\right]}^{T}$, where $\beta_{3}$ is the parameter of interest. The estimating functions are defined as $\psi ={\left[\psi_{\alpha }^{T},\psi_{\beta }^{T}\right]}^{T}$, where $\psi_{\alpha }$ and $\psi_{\beta }$ are estimating functions for the validation and main studies, respectively. Consistent point estimates of α and β (denoted by $\hat{\alpha }$ and $\hat{\beta }$, respectively) can then be obtained by solving the system of estimating equation ψ=0.

Specifically, $\psi_{\alpha }$ is the estimating function obtained by applying regression methods in the VS. If there is only a single measurement for each VS participant, we can apply simple linear regression; if there is more than one measurement, then generalized estimating equations (GEEs) regression can be adopted (Liang & Zeger, 1986). The specific forms of $\psi_{\alpha }$ can be found in Web Appendixes A.1 and A.2, and $\psi_{\beta }=\overset{N}{\underset{i=1}{\sum }}{\hat{X}}_{i}^{T}\Sigma_{i}^{-1}(Y_{i}-{\hat{X}}_{i}\beta )$, where $Y_{i}={\left(Y_{i1},\ldots ,Y_{im_{i}}\right)}^{T}$ is the vector of outcomes for the *i*th subject, $\Sigma_{i}$ is the working variance–covariance matrix, ${\hat{X}}_{i}={\left({\hat{X}}_{i}(t_{i1}),\ldots ,{\hat{X}}_{i}(t_{im_{i}})\right)}^{T}$, ${\hat{X}}_{i}(t_{ij})={\left(1,{\hat{X}}_{i}(t_{ij}),t_{ij},{\hat{X}}_{i}(t_{ij})t_{ij},W_{i}^{T}(t_{ij})\right)}^{T}$, and ${\hat{X}}_{i}(t_{ij})=𝔼\left[X_{i}(t_{ij})|{\tilde{C}}_{i}(t_{ij}),{\tilde{t}}_{ij},{\tilde{W}}_{i}(t_{ij});\alpha \right]$. Note that $N=n_{1}+n_{2}$ in IVS and $N=n_{1}$ in EVS. We can show that the estimators obtained in this way are consistent (Web Appendix A.3).

To obtain valid estimates of $Var(\hat{\beta })$, we must consider the variability induced by estimating α in the VS. To do so, we estimate $Var(\hat{\theta })$ using the sandwich variance formula based upon the estimating functions ψ. The sandwich estimator matrix is given by

(7) $$Var(\hat{\theta })=B{\left(\theta \right)}^{-1}A(\theta ){\left(B{\left(\theta \right)}^{-1}\right)}^{T},$$

where A(θ),B(θ) are square matrices of dimension $\left(p_{\alpha }+p_{\beta }\right)$ with forms A(θ)=Var[ψ] and B(θ)=∂ψ/∂θ, and $p_{\alpha }$ and $p_{\beta }$ are the dimensions for the vector α and β. By evaluating $Var(\hat{\theta })$ at estimated values $\hat{\theta }$, we can estimate the variance of $\hat{\beta }$ by the appropriate diagonal elements of $Var(\hat{\theta })$. The forms for A(θ),B(θ) under both the IVS and EVS designs can be found in Web Appendixes A.1 and A.2.

### 2.4 | Considerations of the time scale for analysis

In practice, we may be interested in using time since the baseline assessment as the time scale in the MS; however, this time scale is not well defined in an EVS. For example, in the EVS of our illustrative example, the only time variable available is age. Derivations in Web Appendix A.5 show that the time scale used in the MEM does not have to be the same as that used in the outcome model for valid application of the proposed method. Thus, we will use age as the time scale in the MEM while using time since baseline as the time scale in the outcome model.

## 3 | SIMULATION STUDY

We conducted a simulation study to investigate the finite sample properties of the proposed method under scenarios arising within an MS/VS design, including both EVS and IVS. The sample size for the MS and VS was set to $n_{1}=5000/1000$ and $n_{2}=500/100$, respectively. VS with both a single measurement and repeated measurements of the exposure validation pairs within subjects was considered. We conducted a 2 × 3 factorial design for the true MEM that generates the data. The first factor denotes whether an interaction exists between surrogate exposure and time in the true MEM, which has two levels: (1) interaction is not present (NI) and (2) interaction is present (IP). The second factor represents the status of an additional covariate W in the true MEM, which has three levels: (1) W is not present (NW); (2) W is present, and correlated with surrogate exposure C (WC); (3) W is present, and uncorrelated with surrogate exposure C (WP). In the text below, we will use levels of these two factors to refer to each scenario for the true MEM: for example, NI-NW represents the scenario where the interaction and W are not present in the true MEM.

The specific forms of the true MEMs for each scenario and their corresponding Corr(c,C) are listed as below, with $\alpha_{0}=1.2,\alpha_{1}=0.7,\alpha_{2}=0.6,\alpha_{3}=0.5,\alpha_{4}=0.4$, when included in the model. Note that the data-generating mechanism in our simulation study is consistent with the specified measurement error framework in Equation (2), which models the mean of the true exposure conditional on the surrogate exposure and other covariates. The classical-type error model can be viewed as a special case of our assumed model and more discussion on their relationship can be found in Web Appendix A.6.

Scenario NI-NW: $c_{i}\left(t_{ik}\right)=\alpha_{0}+\alpha_{1}C_{i}\left(t_{ik}\right)+\epsilon_{i}\left(t_{ik}\right);\text{Corr}(c,C)=0.60$.

Scenario NI-WP: $c_{i}\left(t_{ik}\right)=\alpha_{0}+\alpha_{1}C_{i}\left(t_{ik}\right)+\alpha_{4}W_{i}\left(t_{ik}\right)+\epsilon_{i}\left(t_{ik}\right),\text{Corr}(C,W)=0;\text{Corr}(c,C)=0.65$.

Scenario NI-WC: $c_{i}\left(t_{ik}\right)=\alpha_{0}+\alpha_{1}C_{i}\left(t_{ik}\right)+\alpha_{4}W_{i}\left(t_{ik}\right)+\epsilon_{i}\left(t_{ik}\right),\text{Corr}(C,W)=0.4;\text{Corr}(c,C)=0.57$.

Scenario IP-NW: $c_{i}\left(t_{ik}\right)=\alpha_{0}+\alpha_{1}C_{i}\left(t_{ik}\right)+\alpha_{2}t_{ik}+\alpha_{3}C_{i}\left(t_{ik}\right)t_{ik}+\epsilon_{i}\left(t_{ik}\right);\text{Corr}(c,C)=0.84$.

Scenario IP-WP: $c_{i}\left(t_{ik}\right)=\alpha_{0}+\alpha_{1}C_{i}\left(t_{ik}\right)+\alpha_{2}t_{ik}+\alpha_{3}C_{i}\left(t_{ik}\right)t_{ik}+\alpha_{4}W_{i}\left(t_{ik}\right)+\epsilon_{i}\left(t_{ik}\right),\text{Corr}(C,W)=0;\text{Corr}(c,C)=0.85$.

Scenario IP-WC: $c_{i}\left(t_{ik}\right)=\alpha_{0}+\alpha_{1}C_{i}\left(t_{ik}\right)+\alpha_{2}t_{ik}+\alpha_{3}C_{i}\left(t_{ik}\right)t_{ik}+\alpha_{4}W_{i}\left(t_{ik}\right)+\epsilon_{i}\left(t_{ik}\right),\text{Corr}(C,W)=0.4;\text{Corr}(c,C)=0.84$.

In the illustrative example, Corr(c,C)=0.61, so Scenario NI-NW is the closest case to it. We generated $\left(c_{i}\left(t_{ik}\right),C_{i}\left(t_{ik}\right),t_{ik},W_{i}\left(t_{ik}\right)\right)$ for subjects in both MS and VS at five time points. See Web Appendix B.1 for more details regarding our data generation process. In the MS and the VS with repeated measurements within each subject, data generated at all time points were included $\left(l_{i}=5\right)$. In the VS with a single measurement for each subject, we randomly selected data generated at one time point to use ($l_{i}=1$), as in Liao et al. (2018). Then, we generated exposure history $X_{i}(t_{ij}),Z_{i}(t_{ij})$ from the cumulative average function based on $c_{i}\left(t_{ik}\right),C_{i}\left(t_{ik}\right)$, respectively.

Lastly, we simulated outcome vectors $Y_{i}={\left(Y_{i}\left(t_{i1}\right),\ldots Y_{i}\left(t_{i5}\right)\right)}^{T}∼MVN\left(\mu_{i},\Sigma_{Y}\right)$ for each subject i, with mean values $\mu_{i}={\left(\mu_{i1},\ldots \mu_{i5}\right)}^{T}$ generated based on formula (1), and $\Sigma_{Y}$ following an AR(1) structure with variance $\sigma_{Y}^{2}=1$ and correlation for adjacent elements $\rho_{Y}=0.1$. For true parameters, we fixed $\beta_{0}=0.5$, $\beta_{2}=3$ and $\beta_{4}=1$. Four combinations were considered for exposure-related coefficients $\left(\beta_{1},\beta_{3}):(3,3),(3,0.2),(0.2,3),(0.2,0.2\right)$, leading to Corr(X,Y) ranging from 0.1 to 0.7, to consider a weak and strong impact of the exposure on the outcome.

We applied our proposed method using standard linear regression models in the VS for estimating α, and GEE in the MS for estimating β. When implementing GEE for scenarios with no covariate W, we examined three types of working correlation structure, which models the correlation among the outcomes measured at different occasions for each individual: unstructured, AR(1), and independent, and we found that the bias and coverage probability were invariant to the choice of the correlation structure. Thus, for the remaining scenarios where W was included, we only considered AR(1) for the working correlation. The relative bias in percent, $\left\{100*(\bar{\hat{\beta }}-\beta )/\beta \}(\%\right)$, was calculated to assess the finite sample estimation performance, where $\bar{\hat{\beta }}$ was the average of estimates from 1000 simulated data. The empirical standard error (ESE) of $\hat{\beta }$ was used as the gold standard to assess the performance of estimated sandwich standard error (SE) given in Equation (7). To investigate the bias caused by exposure measurement error, we computed the uncorrected estimates for β ignoring measurement error.

We compared the method performance when the model was correctly specified and when not. In the former case, all variables used to generate the data in the true MEM (6) were also included when fitting this model in the VS as part of the estimation procedure, while in the latter case, the MEM fit in the VS took a different form. We considered two scenarios under MEM misspecification. For scenarios where a covariate, W, was included in the true MEM, the MEM fit in the VS ignored that covariate. For scenarios where an interaction between surrogate exposure and time was present in the true MEM, the MEM fit in the VS failed to include that interaction term.

Since primary interest was in $\beta_{3}$, we only presented results for this parameter. Tables 1–4 display results for the MS/EVS design, as in our illustrative example, when the working correlation was AR(1). All other results can be found in Web Appendix B.2, including those with working unstructured and independence working correlation for the MS/EVS design, and all results for the MS/IVS design. Table 5 provides a summary of all simulation tables.

Overall, if MEM misspecification did not exist when fitting the model, our method performed consistently well under all data-generating mechanisms considered. The relative bias of our corrected method was much smaller than that given by the uncorrected method. Our corrected SE also approximated the ESE very well. The results showed that the corrected estimators’ 95% confidence interval coverage probabilities performed much better than those of the uncorrected estimators, and most were within the nominal 95% coverage bounds of [93.6%, 96.4%] for 1000 replications, with only a few were slightly out of range. If repeated measurements were available in the VS, it could further improve the performance of our approach compared to the case where only one measurement was available per subject (Table 1).

When the MEM was misspecified, it sometimes, but not always, caused bias. When W was correlated with C, it was important to include it when fitting the MEM. Failing to do so led to biased estimators and low 95% coverage probabilities. However, when W and C were uncorrelated, ${\hat{\beta }}_{3}$ was unbiased whether or not W was included in the MEM (Tables 2 and 4). Ignoring the interaction term in the estimation procedure when it was present in the data-generating mechanism also resulted in substantially biased estimators in many scenarios considered (Table 3).

Under all scenarios we investigated, the results after applying our method were comparable between IVS and EVS. However, in practice, IVS, if feasible, is preferable to EVS due to the elimination of the unverifiable transportablity assumption required in the MS/EVS design. In addition, the results were also similar regardless of the specification of the working correlation structure, which indicated the robustness of our method.

## 4 | ILLUSTRATIVE EXAMPLE

We applied our measurement error correction method to assess the impact of long-term exposure to PM_2.5_ in relation to cognitive decline in the NHS Cognitive Cohort, extending the analysis in a previously published paper (Weuve et al., 2012). This paper used the surrogate exposure without considering the measurement error issue, and here we correct for this bias due to such mismeasurement.

### 4.1 | Motivating example

#### 4.1.1 | Assessment of outcome and surrogate exposure

Participants in the NHS Cognitive Cohort were given several cognitive-related tests three times at approximately 2-year intervals, including tests of general cognition, verbal memory, category fluency, working memory, and attention. Based on these test results, an integrated indicator for overall cognition, the global cognitive score, was created and will be used as the outcome variable in our analysis (Web Appendix C). A more detailed description of the study population and outcome assessment has been previously published (Weuve et al., 2012).

Geographic Information System (GIS)-based spatiotemporal models were used to estimate exposure to PM_2.5_ for women residing in the contiguous United States. These models and their use in evaluating chronic PM exposures among the NHS cohort are described in detail elsewhere (Puett et al., 2008, 2009; Paciorek et al., 2009; Weuve et al., 2012; Yanosky et al., 2008, 2014). Briefly, monitor data on PM_2.5_ were obtained from the US Environmental Protection Agency’s Air Quality System. Then, a generalized additive model was constructed to predict monthly PM_2.5_ concentrations based on the monitor data, research studies, and other GIS-based covariates, such as population density, distance to nearest roads, and weather variables (Hart et al., 2015; Kioumourtzoglou et al., 2014). These data were then linked to each person-visit of follow-up by longitude and latitude of the last recorded nurse’s address, usually within 2 years. The surrogate exposure was then calculated as average month-specific PM_2.5_ exposure from 1989 through the month before baseline cognitive assessment (Web Appendix C).

#### 4.1.2 | Exposure validation study

Personal exposure to PM_2.5_ of ambient origin, the true exposure, was available from studies performed in four U.S. cities between 1999 and 2002 among 108 adults. ${SO}_{4}^{2-}$ was used to estimate it (Kioumourtzoglou et al., 2014): ${PM}_{\text{pers. of ambient origin}}=\frac{{SO}_{4}^{2-}\text{pers.}}{{SO}_{4}^{2-}\text{ambient}}{PM}_{\text{ambient}}$, because indoor sources were negligible and ${SO}_{4}^{2-}$ had similar spatial homogeneity as PM_2.5_ (Sarnat et al., 2002).

Surrogate exposure for this group was obtained in the same way as described above. Paired information on true and surrogate exposure in the VS allowed us to estimate the relationship between them through the MEM. In this VS, around 41% people have measurements at more than one time point (Web Appendix C). To fully use the available data, we derived the generalized version of our correction method to accommodate this situation, which can be found in Web Appendix A.2.

#### 4.1.3 | Potential confounders

Potential confounders are those that may affect the extent to which cognitive function declines as time passes at different levels of PM_2.5_ exposure, such as education (registered nurse degree, bachelor’s degree, or advanced graduate degree), husband’s education (high school diploma or less, college degree, advanced graduate degree, or other), physical activity (cumulative average of responses to four to seven questionnaires from 1986 through initial cognitive assessment; initially measured as MET-hours/week, then coded as a categorical variable in quartiles) and alcohol consumption (cumulative average of responses to five to eight questionnaires from 1986 through initial cognitive assessment, coded as none, up to 1 drink/week, 2–6 drinks/week, or ≥1 drink/d). They were available in the MS (Web Appendix C), but were not in the EVS. This posed a challenge to application of our correction method, and will be discussed further below.

### 4.2 | Results

The sample size of participants in the MS and VS were 19, 002 and 108, respectively. Among all 19, 002 participants, 12, 303 had fully observed outcomes (i.e., the total number of cognitive assessments was 3) and 6699 had missing measurements (i.e., the total number of cognitive assessments was less than 3). Here, we assumed that the missing mechanism was missing completely at random (MCAR), as comparison of covariates between these two groups of people suggested that they were very similar (Table S16). As mentioned above in Section 4.1, one challenge in correcting for bias of effect estimates due to mismeasured exposure here was that the EVS had limited covariate data; in particular, only data on date of exposure assessment and age. Recall that when variables are associated with outcome but are not correlated with exposure, they are not confounders, and the exposure effect can be estimated validly without data in such variables. With this in mind, we first fitted a partial model and a full model in the MS data to identify which covariates were likely to be confounders, hoping to remove unnecessary variables from the outcome model, so that they would not be needed in the MEM (2). For the time-related variables in the MS, we used time since baseline as time scale to be consistent with the original study (Weuve et al., 2012), so that the results were comparable.

Since our main interest lied in assessing the difference in 2-year changes in cognitive score per 10 *μ*g/m^3^ increase in PM_2.5_, as was the focus of the original paper, we determined that a variable was not a confounder and could be validly removed from the main model if, in the partial model, the estimate of the parameter of interest, here ${\hat{\beta }}_{3}$, did not change more than 10% compared to its estimated value from the full model. Using this criterion, the fitted model suggested that education, husband’s education, physical activity, and alcohol consumption were not confounders, as the change in ${\hat{\beta }}_{3}$ was only around 3%. This was not surprising given that residence-based exposure is “exogenously” determined and not likely to be influenced by personal characteristics (Weisskopf & Webster, 2017).

When fitting the MEM, we used surrogate exposure, age, interaction between them as covariates, true exposure as the outcome, and an independence matrix as the working correlation matrix. When fitting the outcome model, we used time since baseline, exposure history function and its interaction with time, baseline age and its interaction with time as covariates, cognitive score as the outcome, and an unstructured matrix as the working matrix. We also tried fitting the MS data using AR(1), and the result was very similar. The estimated value of the parameter of interest after applying our method was −0.027 (95% CI (−0.059, 0.005)); compared to −0.018 (95% CI (−0.034, −0.001)) in the previous paper, a 60% greater effect. Our result can be interpreted as the 2-year decline on global cognitive score is 0.027 units worse per 10 *μ*g/m^3^ increase in PM_2.5_ exposure. To put this finding into perspective, effects of this magnitude, based on the measurement error-corrected analysis, are about 2/3 of those found in our data associated with each additional year of aging: 0.044 (95% CI, −0.047 to −0.040) units per 1 year older, hence quite important from a public health perspective.

We also graphed the cognitive score trajectories over time from the original and the measurement error-corrected results. As shown in Figure S1 (Web Appendix C), the rate of decline of the cognitive score for a given level of PM_2.5_ and baseline age was higher when using the measurement error adjusted estimates, compared to the estimated trajectory ignoring measurement error.

It is worth mentioning that the localized error assumption when age is used as the time scale can be tested empirically using the VS data. Here, we adopted the sequential ANOVA test, and it suggested that given current surrogate exposure and age, adding average of previous surrogate exposure to the model was not significantly associated with the current true exposure (*p*-value = 0.31). The transportability assumption cannot be empirically verified in an MS/EVS design. Nevertheless, we provide some justification for similarity between the two groups of people by looking at distribution of important variables in Table S15; in particular, age and monthly spatial-temporal predicted PM_2.5_.

## 5 | DISCUSSION

Motivated by an investigation of the impact of PM_2.5_ exposure on cognitive decline in Cognitive Cohort of the NHS, we proposed a new method to correct for bias in exposure effect estimates due to a mismeasured exposure history in longitudinal studies with continuous outcomes. Our results showed that the effect of PM_2.5_ exposure on 2-year cognitive decline has been underestimated by 60%. Although the motivating study had a MS/EVS design and the exposure history function used was a moving average, this method is generalizable to studies with MS/IVS design, and those derivations can be found in Web Appendix A.1, and for which other functions of the exposure history are of interest. A major contribution of this method is that it can be applied to validation studies with both a single measurement and repeated measurements of the exposure validation pairs within subjects. In addition, it is robust to misspecification of the working correlation structure and allows for the use of different time scales in the MS and VS models, a common scenario in the MS/VS design.

As discussed in Section 2.2, the surrogacy assumption is needed for our proposed method. In MV/EVS design (our case here), it must be assumed; while in MS/IVS design, it can be empirically verified. For IVS participants, we can include surrogate exposure ${\tilde{C}}_{i}(t_{ij})$ in the outcome model for $𝔼\left[Y_{i}(t_{ij})|\tilde{c_{i}}(t_{ij}),{\tilde{t}}_{ij},{\tilde{W}}_{i}(t_{ij})\right]$, and test whether their coefficients are zero in the model.

Besides, we assume that the measurement error process is “localized.” To put this in the context of our illustrative example, this assumption implied that the personal exposure to PM_2.5_ depends only on the current residential address-based exposure, other variables at the current time, and the current time itself. In other words, conditional on all variables at the current time, the personal exposure to PM_2.5_ is independent of these variables in the past. This assumption is plausible in the environmental exposure setting, and can be tested in a VS that includes longitudinal data of the surrogate exposure. For example, if the longitudinal surrogate exposures $C_{i}(t_{ij}),\ldots ,C(t_{i(j-m)})$ are available for the *i*th participant, where m is the lag between the current and earliest time point available, we can include $C_{i}(t_{ij}),\ldots ,C(t_{i(j-m)})$ in the MEM for $𝔼\left[c_{i}(t_{ik})|C_{i}(t_{i1}),\ldots ,C_{i}(t_{ij}),t_{i1},\ldots ,t_{ij},W_{i}(t_{i1}),\ldots ,W_{i}(t_{ij})\right]$ and test whether the coefficients of $C_{i}(t_{i(j-1)}),\ldots ,C(t_{i(j-m)})$ are zero in the model. Since there is typically colinearity between $C_{i}(t_{ij}),\ldots ,C(t_{i(j-m)})$ and validation studies are small, instead of including $C_{i}(t_{ij}),\ldots ,C(t_{i(j-m)})$ in the same MEM, we can first fit the model with only $C_{ij},t_{ij},W_{i}(t_{ij})$ as covariates, and in the second step, we can use the residuals from the first step as the outcome and include the cumulative average of $C_{i}(t_{i(j-1)}),\ldots ,C(t_{i(j-m)})$ and that of $W_{i}(t_{i(j-1)}),\ldots ,W_{i}(t_{i(j-m)})$ as covariates to jointly test whether their coefficients are zero. If $C_{i}(t_{i(j-1)}),\ldots ,C(t_{i(j-m)})$ is found to be uncorrelated with $c_{ij}$ conditional on $C_{ij}$, it is typically reasonable to assume that $C(t_{i1}),\ldots ,C(t_{i(j-m-1)})$ are also uncorrelated with $c_{ij}$. Moreover, if the localized error assumption is violated and longitudinal data are available in the VS, with no change in the theory, the MEM can also include these past covariates.

It is common for longitudinal data to have missing observations in practice. Our approach allows for an individual-specific number of measurements $m_{i}$ for the *i*th individual, rather than requiring a common number of measurements. If the missing mechanism is MCAR, our approach will provide valid estimation and inferences. If missing mechanism is missing at random (MAR) or missing not at random (MNAR), our approach may lead to biased results as it is based on the GEE approach and GEE may result in biased estimates under MAR and MNAR (Copas & Seaman, 2010; Touloumi et al., 2001). Under certain MAR pattern, statistical approaches have been developed to reduce or eliminate bias (Chen et al., 2021; da Silva et al., 2019; Robins et al., 1995; Sun et al., 2018). How to extend our current model to handle missing data under MAR mechanism is a complex topic and one for our future research.

In this paper, in the MS/IVS design, when estimating coefficients for the outcome model, we used the estimated exposure history function ${\hat{X}}_{i}$, rather than true $X_{i}$, for participants in the IVS. In the MS/IVS design, several alternative “regression calibration”-type estimators have been proposed (Spiegelman et al., 2001; Thurston et al., 2005). In future research, these could be compared for asymptotic relative efficiency to further refine the best approaches in the longitudinal data analysis setting where $\beta_{3}$ is the parameter of interest. Moreover, we assume a linear relationship between true and surrogate exposure in the MEM in the simulation study, whereas the theoretical derivation in Section 2 does not require this linearity and a nonlinear association could also be considered in analysis should the data suggest this. Besides, we considered marginal models with the GEE approach here. With an identity or log link function, the estimates from the marginal model equal those of the conditional model (Ritz & Spiegelman, 2004). In the future, we will extend this method to models where this equivalence does not hold; in particular, in logistic regression. Measurement error correction methods for causal inference models such as inverse probability weighting (Robins et al., 2000) are also an important area for future research.

## Supplementary Material

Supplementary Data

## Figures and Tables

**TABLE 1:** Bias (%), empirical standard error (ESE), estimated sandwich standard error (SE), and coverage probability (CP) of 95% confidence interval for the interaction effect estimate ${\hat{\beta }}_{3}$ based on 1000 simulations, under the EVS Scenario NI-NW in the true MEM. Working correlation specified as AR(1) in the GEE analysis for the outcome model. $l_{i}$ denotes the number of measurements in VS.

$\beta_{1}$	$\beta_{3}$	Sample size $n_{1},n_{2}$	Fitting method		
			Uncorrected method Bias(%) (ESE) CP	Proposed, $l_{i}=1$ Bias (%) (ESE) (SE) CP	Proposed, $l_{i}=5$ Bias(%) (ESE) (SE) CP
3	3	1000, 100	−29.97 (0.10) 0.00	2.11 (0.46) (0.45) 0.94	0.89 (0.25) (0.23) 0.95
		500	−30.20 (0.10) 0.00	−0.10 (0.23) (0.23) 0.94	0.16 (0.17) (0.17) 0.95
		5000, 100	−29.96 (0.04) 0.00	3.21 (0.48) (0.44) 0.95	0.06 (0.19) (0.19) 0.95
		500	−30.02 (0.04) 0.00	0.30 (0.19) (0.19) 0.95	0.04 (0.10) (0.10) 0.95
3	0.2	1000, 100	−29.73 (0.02) 0.20	2.48 (0.04) (0.04) 0.90	1.91 (0.03) (0.03) 0.95
		500	−30.74 (0.02) 0.17	−0.88 (0.03) (0.03) 0.95	0.47 (0.03) (0.03) 0.95
		5000, 100	−29.85 (0.01) 0.00	3.39 (0.04) (0.03) 0.95	−0.24 (0.02) (0.02) 0.95
		500	−29.97 (0.01) 0.00	0.38 (0.02) (0.02) 0.95	0.05 (0.01) (0.01) 0.95
0.2	3	1000, 100	−29.98 (0.09) 0.00	2.09 (0.46) (0.44) 0.94	0.83 (0.24) (0.22) 0.95
		500	−30.16 (0.09) 0.00	−0.05 (0.22) (0.22) 0.94	0.14 (0.15) (0.15) 0.95
		5000, 100	−29.96 (0.04) 0.00	3.21 (0.48) (0.44) 0.95	0.17 (0.19) (0.19) 0.95
		500	−30.02 (0.04) 0.00	0.30 (0.19) (0.19) 0.90	0.05 (0.10) (0.10) 0.95
0.2	0.2	1000, 100	−29.97 (0.01) 0.00	2.08 (0.03) (0.03) 0.95	1.09 (0.02) (0.02) 0.95
		500	−30.25 (0.01) 0.00	−0.19 (0.02) (0.02) 0.95	0.12 (0.01) (0.01) 0.94
		5000, 100	−29.89 (0.00) 0.00	3.31 (0.03) (0.03) 0.95	−0.03 (0.01) (0.01) 0.94
		500	−30.00 (0.00) 0.00	0.32 (0.01) (0.01) 0.96	0.15 (0.01) (0.01) 0.95

**TABLE 2:** Bias (%), empirical standard error (ESE), estimated sandwich standard error (SE), and coverage probability (CP) of 95% confidence interval for the interaction effect estimate ${\hat{\beta }}_{3}$ based on 1000 simulations, under the EVS Scenario NI-WP and NI-WC in the true MEM. Working correlation specified as AR(1) in the GEE analysis for the outcome model.

$\beta_{1}$	$\beta_{3}$	Sample size $n_{1},n_{2}$	Fitting method		
			Uncorrected method Bias (%) (ESE) CP	Proposed, with W Bias (%) (ESE) (SE) CP	Proposed, no W Bias (%) (ESE) (SE) CP
Results for NI-WP					
3	3	1000, 100	−30.04 (0.11) 0.00	−0.47 (0.39) (0.37) 0.92	1.14 (0.50) (0.48) 0.93
		500	−30.04 (0.12) 0.00	0.13 (0.21) (0.21) 0.95	0.70 (0.27) (0.26) 0.93
		5000, 100	−30.02 (0.05) 0.00	−0.51 (0.36) (0.35) 0.92	1.76 (0.47) (0.46) 0.94
		500	−30.03 (0.05) 0.00	−0.15 (0.17) (0.17) 0.95	0.23 (0.22) (0.21) 0.94
3	0.2	1000, 100	−30.26 (0.02) 0.24	−0.09 (0.04) (0.04) 0.94	0.83 (0.05) (0.04) 0.94
		500	−30.16 (0.02) 0.25	0.03 (0.03) (0.03) 0.95	0.55 (0.04) (0.04) 0.95
		5000, 100	−30.12 (0.01) 0.00	−0.25 (0.03) (0.03) 0.94	1.63 (0.03) (0.03) 0.95
		500	−30.03 (0.01) 0.00	−0.18 (0.02) (0.02) 0.94	0.24 (0.02) (0.02) 0.94
0.2	3	1000, 100	−30.03 (0.10) 0.00	−0.50 (0.38) (0.37) 0.92	1.16 (0.50) (0.47) 0.92
		500	−30.04 (0.10) 0.00	0.13 (0.20) (0.20) 0.95	0.70 (0.26) (0.25) 0.93
		5000, 100	−30.01 (0.04) 0.00	−0.53 (0.36) (0.35) 0.92	1.77 (0.47) (0.46) 0.94
		500	−30.04 (0.05) 0.00	−0.16 (0.17) (0.16) 0.95	0.22 (0.21) (0.21) 0.94
0.2	0.2	1000, 100	−30.09 (0.01) 0.00	−0.42 (0.03) (0.03) 0.93	1.08 (0.04) (0.03) 0.93
		500	−30.23 (0.01) 0.00	−0.03 (0.02) (0.02) 0.96	0.42 (0.02) (0.02) 0.94
		5000, 100	−30.01 (0.00) 0.00	−0.38 (0.02) (0.02) 0.93	1.81 (0.03) (0.03) 0.94
		500	−30.06 (0.00) 0.00	−0.20 (0.01) (0.01) 0.95	0.20 (0.02) (0.01) 0.94
Results for NI-WC					
3	3	1000, 100	−17.49 (0.11) 0.01	−0.50 (0.33) (0.32) 0.92	−3.39 (0.38) (0.36) 0.88
		500	−17.53 (0.12) 0.01	0.08 (0.18) (0.18) 0.95	−3.59 (0.21) (0.20) 0.88
		5000, 100	−17.50 (0.05) 0.00	−0.78 (0.30) (0.30) 0.92	−3.16 (0.36) (0.35) 0.89
		500	−17.50 (0.05) 0.00	−0.17 (0.15) (0.14) 0.95	−3.86 (0.17) (0.16) 0.83
3	0.2	1000, 100	−25.98 (0.02) 0.35	−0.65 (0.03) (0.03) 0.94	−13.30 (0.03) (0.03) 0.82
		500	−25.98 (0.02) 0.38	−0.10 (0.03) (0.03) 0.94	−13.46 (0.03) (0.03) 0.83
		5000, 100	−25.92 (0.01) 0.00	−1.05 (0.02) (0.02) 0.94	−13.03 (0.02) (0.02) 0.70
		500	−25.81 (0.01) 0.00	−0.29 (0.01) (0.01) 0.94	−13.54 (0.02) (0.01) 0.55
0.2	3	1000, 100	−16.97 (0.10) 0.00	−0.49 (0.33) (0.32) 0.92	−2.78 (0.38) (0.36) 0.89
		500	−17.01 (0.10) 0.00	0.08 (0.17) (0.17) 0.95	−2.98 (0.20) (0.19) 0.89
		5000, 100	−16.97 (0.04) 0.00	−0.75 (0.30) (0.30) 0.92	−2.54 (0.36) (0.35) 0.90
		500	−16.98 (0.05) 0.00	−0.17 (0.15) (0.14) 0.95	−3.26 (0.16) (0.16) 0.86
0.2	0.2	1000, 100	−19.54 (0.01) 0.04	−0.54 (0.02) (0.02) 0.92	−5.78 (0.03) (0.03) 0.86
		500	−19.70 (0.01) 0.04	−0.08 (0.01) (0.01) 0.95	−6.14 (0.02) (0.02) 0.85
		5000, 100	−19.45 (0.00) 0.00	−0.72 (0.02) (0.02) 0.94	−5.43 (0.02) (0.02) 0.85
		500	−19.49 (0.00) 0.00	−0.23 (0.01) (0.01) 0.94	−6.19 (0.01) (0.01) 0.75

**TABLE 3:** Bias (%), empirical standard error (ESE), estimated sandwich standard error (SE), and coverage probability (CP) of 95% confidence interval for the interaction effect estimate ${\hat{\beta }}_{3}$ based on 1000 simulations, under the EVS Scenario IP-NW in the true MEM. Working correlation specified as AR(1) in the GEE analysis for the outcome model.

$\beta_{1}$	$\beta_{3}$	Sample size $n_{1},n_{2}$	Fitting method		
			Uncorrected method Bias (%) (ESE) CP	Proposed, with interaction Bias (%) (ESE) (SE) CP	Proposed, no interaction Bias (%) (ESE) (SE) CP
3	3	1000, 100	546.08 (0.34) 0.00	0.15 (0.11) (0.10) 0.94	43.04 (0.29) (0.26) 0.00
		500	545.71 (0.34) 0.00	−0.04 (0.05) (0.05) 0.95	42.94 (0.14) (0.13) 0.00
		5000, 100	545.18 (0.15) 0.00	−0.10 (0.10) (0.10) 0.92	42.90 (0.26) (0.25) 0.00
		500	545.56 (0.15) 0.00	0.00 (0.04) (0.04) 0.97	42.83 (0.12) (0.12) 0.00
3	0.2	1000, 100	1068.75 (0.04) 0.00	0.77 (0.02) (0.02) 0.93	145.02 (0.03) (0.03) 0.00
		500	1067.82 (0.04) 0.00	−0.08 (0.01) (0.01) 0.95	144.85 (0.02) (0.02) 0.00
		5000, 100	1067.25 (0.02) 0.00	0.21 (0.02) (0.02) 0.93	144.78 (0.03) (0.03) 0.00
		500	1067.67 (0.02) 0.00	0.03 (0.01) (0.01) 0.95	144.82 (0.01) (0.01) 0.00
0.2	3	1000, 100	511.25 (0.34) 0.00	0.12 (0.09) (0.09) 0.94	36.36 (0.27) (0.25) 0.00
		500	510.90 (0.33) 0.00	−0.03 (0.05) (0.05) 0.95	36.26 (0.13) (0.12) 0.00
		5000, 100	510.37 (0.15) 0.00	−0.11 (0.09) (0.09) 0.92	36.23 (0.25) (0.24) 0.00
		500	510.75 (0.14) 0.00	−0.00 (0.04) (0.04) 0.96	36.15 (0.11) (0.11) 0.00
0.2	0.2	1000, 100	555.58 (0.02) 0.00	0.18 (0.01) (0.01) 0.93	45.33 (0.02) (0.02) 0.00
		500	554.93 (0.02) 0.00	−0.07 (0.00) (0.00) 0.95	45.20 (0.01) (0.01) 0.00
		5000, 100	554.51 (0.01) 0.00	−0.09 (0.01) (0.01) 0.91	45.17 (0.02) (0.02) 0.00
		500	554.92 (0.01) 0.00	−0.02 (0.00) (0.00) 0.96	45.13 (0.01) (0.01) 0.00

**TABLE 4:** Bias (%), empirical standard error (ESE), estimated sandwich standard error (SE), and coverage probability (CP) of 95% confidence interval for the interaction effect estimate ${\hat{\beta }}_{3}$ based on 1000 simulations, under the EVS Scenario IP-WP and IP-WC in the true MEM. Working correlation specified as AR(1) in the GEE analysis for the outcome model.

$\beta_{1}$	$\beta_{3}$	Sample size $n_{1},n_{2}$	Fitting method		
			Uncorrected method Bias (%) (ESE) CP	Proposed, with W Bias (%) (ESE) (SE) CP	Proposed, no W Bias (%) (ESE) (SE) CP
Results for IP-WP					
3	3	1000, 100	544.12 (0.34) 0.00	−0.14 (0.10) (0.10) 0.94	−0.22 (0.11) (0.11) 0.93
		500	544.83 (0.33) 0.00	−0.10 (0.05) (0.05) 0.94	−0.09 (0.06) (0.06) 0.94
		5000, 100	544.71 (0.15) 0.00	−0.02 (0.10) (0.10) 0.93	−0.12 (0.11) (0.11) 0.93
		500	544.37 (0.15) 0.00	0.02 (0.05) (0.05) 0.95	−0.01 (0.05) (0.05) 0.95
3	0.2	1000, 100	1063.03 (0.04) 0.00	0.10 (0.02) (0.02) 0.92	−0.11 (0.02) (0.02) 0.93
		500	1064.06 (0.04) 0.00	−0.31 (0.01) (0.01) 0.94	−0.29 (0.01) (0.01) 0.94
		5000, 100	1064.02 (0.02) 0.00	0.48 (0.02) (0.02) 0.92	0.03 (0.02) (0.02) 0.91
		500	1063.72 (0.02) 0.00	0.16 (0.01) (0.01) 0.95	0.08 (0.01) (0.01) 0.95
0.2	3	1000, 100	509.55 (0.34) 0.00	−0.16 (0.09) (0.09) 0.93	−0.22 (0.10) (0.10) 0.94
		500	510.23 (0.32) 0.00	−0.09 (0.05) (0.05) 0.94	−0.08 (0.05) (0.05) 0.94
		5000, 100	510.12 (0.14) 0.00	−0.05 (0.09) (0.09) 0.93	−0.13 (0.10) (0.10) 0.93
		500	509.77 (0.15) 0.00	0.01 (0.04) (0.04) 0.95	−0.02 (0.05) (0.05) 0.94
0.2	0.2	1000, 100	553.01 (0.02) 0.00	−0.13 (0.01) (0.01) 0.93	−0.20 (0.01) (0.01) 0.93
		500	553.67 (0.02) 0.00	−0.18 (0.00) (0.00) 0.94	−0.17 (0.00) (0.00) 0.95
		5000, 100	553.73 (0.01) 0.00	0.01 (0.01) (0.01) 0.93	−0.10 (0.01) (0.01) 0.93
		500	553.31 (0.01) 0.00	0.01 (0.00) (0.00) 0.95	−0.02 (0.00) (0.00) 0.95
Results for IP-WC					
3	3	1000, 100	556.92 (0.34) 0.00	−0.18 (0.10) (0.10) 0.93	−1.71 (0.11) (0.11) 0.90
		500	557.58 (0.33) 0.00	−0.11 (0.05) (0.05) 0.95	−1.65 (0.06) (0.06) 0.86
		5000, 100	557.49 (0.15) 0.00	−0.04 (0.10) (0.10) 0.93	−1.67 (0.11) (0.10) 0.89
		500	557.15 (0.15) 0.00	0.01 (0.05) (0.05) 0.95	−1.52 (0.05) (0.05) 0.85
3	0.2	1000, 100	1066.38 (0.04) 0.00	−0.06 (0.02) (0.02) 0.92	−4.25 (0.02) (0.02) 0.91
		500	1067.25 (0.04) 0.00	−0.33 (0.01) (0.01) 0.95	−4.56 (0.01) (0.01) 0.86
		5000, 100	1067.20 (0.02) 0.00	0.39 (0.02) (0.02) 0.93	−4.23 (0.02) (0.02) 0.88
		500	1066.92 (0.02) 0.00	0.13 (0.01) (0.01) 0.95	−4.07 (0.01) (0.01) 0.85
0.2	3	1000, 100	522.67 (0.33) 0.00	−0.19 (0.09) (0.09) 0.93	−1.53 (0.10) (0.10) 0.90
		500	523.32 (0.32) 0.00	−0.10 (0.05) (0.05) 0.94	−1.44 (0.05) (0.05) 0.86
		5000, 100	523.23 (0.14) 0.00	−0.06 (0.09) (0.09) 0.93	−1.48 (0.10) (0.09) 0.89
		500	522.88 (0.15) 0.00	0.00 (0.04) (0.04) 0.95	−1.33 (0.04) (0.04) 0.85
0.2	0.2	1000, 100	564.91 (0.02) 0.00	−0.16 (0.01) (0.01) 0.93	−1.55 (0.01) (0.01) 0.91
		500	565.52 (0.02) 0.00	−0.18 (0.00) (0.00) 0.94	−1.58 (0.00) (0.00) 0.89
		5000, 100	565.62 (0.01) 0.00	0.00 (0.01) (0.01) 0.93	−1.50 (0.01) (0.01) 0.91
		500	565.20 (0.01) 0.00	0.01 (0.00) (0.00) 0.95	−1.37 (0.00) (0.00) 0.88

**TABLE 5:** Summary of all simulation result tables. Scenarios under true MEM are as defined in Section 3. We only considered AR(1) as the working correlation in scenarios where W was included; because when we applied our method for scenarios where W was not present, we found that the bias and coverage probability were invariant to the choice of the correlation structure.

Validation study	True MEM	Working correlation for the outcome model		
		AR(1)	Unstructured	Independence
EVS	NI-NW	Main 1	Supplement 5	Supplement 7
	NI-WP; NI-WC	Main 2	—	—
	IP-NW	Main 3	Supplement 6	Supplement 8
	IP-WP; IP-WC	Main 4	—	—
IVS	NI-NW	Supplement 1	Supplement 9	Supplement 11
	NI-WP; NI-WC	Supplement 2	—	—
	IP-NW	Supplement 3	Supplement 10	Supplement 12
	IP-WP; IP-WC	Supplement 4	—	—

## Data Availability

The data that support the findings in this paper are not publicly available due to privacy or ethical restrictions.
