# Supplementary material for: Correcting for bias due to mismeasured exposure history in longitudinal studies with continuous outcomes
Source: Biometrics. Author manuscript; Available in PMC 2024 Sep 1. (PMC11214728; doi:10.1111/biom.13877)
Supplement: Supplementary Data [file NIHMS1926016-supplement-Supplementary_Data.pdf]

# Supporting Information for Correcting for bias due to mismeasured exposure history in longitudinal studies with continuous outcomes by Jiachen Cai, Ning Zhang, Xin Zhou, Donna Spiegelman, and Molin Wang

## Web Appendix A Mathematical Derivations

### A.1 Fully Independent Validation Data

As defined in the manuscript, $\boldsymbol{\psi}_{\boldsymbol{\beta}} = \sum_{i=1}^{N} \widehat{\mathbf{X}}_i^T \boldsymbol{\Sigma}_i^{-1}(\mathbf{Y}_i - \widehat{\mathbf{X}}_i\boldsymbol{\beta})$, where $\mathbf{Y}_i = (Y_{i1}, ...Y_{im_i})^T$ is the vector of outcomes for the $i$th subject, $\boldsymbol{\Sigma}_i$ is the working variance-covariance matrix, $\widehat{\mathbf{X}}_i = (\widehat{\mathbf{X}}_i(t_{i1}), ..., \widehat{\mathbf{X}}_i(t_{im_i}))^T$, $\widehat{\mathbf{X}}_i(t_{ij}) = (1, \widehat{X_i}(t_{ij}), t_{ij}, \widehat{X_i}(t_{ij})t_{ij}, \mathbf{W}_i^T(t_{ij}))^T$, and $\widehat{X_i}(t_{ij}) = E[X_i(t_{ij})|\widetilde{C}_i(t_{ij}), \widetilde{t}_{ij}, \widetilde{\mathbf{W}}_i(t_{ij}); \boldsymbol{\alpha}]$. Note that $N = n_1 + n_2$ for the main and internal validation study design, and $N = n_1$ for the main and external validation study design. Assume that for the $i$th person in the validation study, $\mathbf{d}_i(t_i) = (1, C_i(t_i), t_i, C_i(t_i)t_i, W_i(t_i))^T$, $i = n_1 + 1, ..., n_1 + n_2$. We define $\boldsymbol{\psi}_{\boldsymbol{\alpha}} = \sum_{i=n_1+1}^{n_1+n_2} \mathbf{d}_i(c_i - \mathbf{d}_i^T\boldsymbol{\alpha})$. Also, $\boldsymbol{\theta} = [\boldsymbol{\alpha}^T, \boldsymbol{\beta}^T]^T$ and $\boldsymbol{\psi} = (\boldsymbol{\psi}_{\boldsymbol{\alpha}}^T, \boldsymbol{\psi}_{\boldsymbol{\beta}}^T)^T$.

For the estimate $\widehat{\boldsymbol{\theta}}=(\widehat{\boldsymbol{\alpha}}^T, \widehat{\boldsymbol{\beta}}^T)^T$, we have

$$\mathbf{0} = \boldsymbol{\psi}(\widehat{\boldsymbol{\theta}}) \approx \boldsymbol{\psi}(\boldsymbol{\theta}) + \frac{\partial \boldsymbol{\psi}}{\partial \boldsymbol{\theta}}(\widehat{\boldsymbol{\theta}} - \boldsymbol{\theta}).$$

Then,

$$\widehat{\boldsymbol{\theta}} - \boldsymbol{\theta} \approx \left(-\frac{\partial \boldsymbol{\psi}}{\partial \boldsymbol{\theta}}\right)^{-1} \boldsymbol{\psi}(\boldsymbol{\theta}).$$

The variance of $\widehat{\boldsymbol{\theta}}$ can be derived as

$$\mathrm{Var}(\widehat{\boldsymbol{\theta}}) \approx \left(\frac{\partial \boldsymbol{\psi}}{\partial \boldsymbol{\theta}}\right)^{-1} \mathrm{Var}(\boldsymbol{\psi}(\boldsymbol{\theta})) \left(\left(\frac{\partial \boldsymbol{\psi}}{\partial \boldsymbol{\theta}}\right)^{-1}\right)^T,$$

and it can be estimated by plugging in estimated values $\widehat{\boldsymbol{\theta}}$.

Define $\mathbf{A}(\boldsymbol{\theta}) = \text{Var}(\boldsymbol{\psi}(\boldsymbol{\theta})) = \begin{bmatrix} \text{Var}(\boldsymbol{\psi}_{\boldsymbol{\alpha}}) & \text{Cov}(\boldsymbol{\psi}_{\boldsymbol{\alpha}}, \boldsymbol{\psi}_{\boldsymbol{\beta}}) \\ \text{Cov}(\boldsymbol{\psi}_{\boldsymbol{\beta}}, \boldsymbol{\psi}_{\boldsymbol{\alpha}}) & \text{Var}(\boldsymbol{\psi}_{\boldsymbol{\beta}}) \end{bmatrix}$. For either an external validation study or an internal validation study, we have

$$\text{Var}(\boldsymbol{\psi}_{\boldsymbol{\alpha}}) = \sum_{i=n_1+1}^{n_1+n_2} (c_i - \mathbf{d}_i^T \boldsymbol{\alpha})^2 \mathbf{d}_i \mathbf{d}_i^T.$$

In an external validation study, because main study participants and validation study participants are different people, $\text{Cov}(\boldsymbol{\psi}_{\boldsymbol{\alpha}}, \boldsymbol{\psi}_{\boldsymbol{\beta}}) = \text{Cov}(\boldsymbol{\psi}_{\boldsymbol{\beta}}, \boldsymbol{\psi}_{\boldsymbol{\alpha}})^T = \mathbf{0}$, and

$$\text{Var}(\boldsymbol{\psi}_{\boldsymbol{\beta}}) = \sum_{i=1}^{n_1} \widehat{\mathbf{X}}_i^T \boldsymbol{\Sigma}_i^{-1} (\mathbf{Y}_i - \widehat{\mathbf{X}}_i \boldsymbol{\beta})(\mathbf{Y}_i - \widehat{\mathbf{X}}_i \boldsymbol{\beta})^T \boldsymbol{\Sigma}_i^{-1} \widehat{\mathbf{X}}_i.$$

In an internal validation study,

$$\text{Cov}(\boldsymbol{\psi}_{\boldsymbol{\alpha}}, \boldsymbol{\psi}_{\boldsymbol{\beta}}) = \sum_{i=n_1+1}^{n_1+n_2} (c_i - \mathbf{d}_i^T \boldsymbol{\alpha}) \mathbf{d}_i (\mathbf{Y}_i - \widehat{\mathbf{X}}_i \boldsymbol{\beta})^T \boldsymbol{\Sigma}_i^{-1} \widehat{\mathbf{X}}_i.$$

By symmetry, we can also obtain $\text{Cov}(\boldsymbol{\psi}_{\boldsymbol{\beta}}, \boldsymbol{\psi}_{\boldsymbol{\alpha}})$. And we also have,

$$\text{Var}(\boldsymbol{\psi}_{\boldsymbol{\beta}}) = \sum_{i=1}^{n_1+n_2} \widehat{\mathbf{X}}_i^T \boldsymbol{\Sigma}_i^{-1} (\mathbf{Y}_i - \widehat{\mathbf{X}}_i \boldsymbol{\beta})(\mathbf{Y}_i - \widehat{\mathbf{X}}_i \boldsymbol{\beta})^T \boldsymbol{\Sigma}_i^{-1} \widehat{\mathbf{X}}_i.$$

Define $\mathbf{B}(\boldsymbol{\theta}) = \frac{\partial \boldsymbol{\psi}}{\partial \boldsymbol{\theta}} = \begin{bmatrix} \frac{\partial \boldsymbol{\psi}_{\boldsymbol{\alpha}}}{\partial \boldsymbol{\alpha}} & \frac{\partial \boldsymbol{\psi}_{\boldsymbol{\alpha}}}{\partial \boldsymbol{\beta}} \\ \frac{\partial \boldsymbol{\psi}_{\boldsymbol{\beta}}}{\partial \boldsymbol{\alpha}} & \frac{\partial \boldsymbol{\psi}_{\boldsymbol{\beta}}}{\partial \boldsymbol{\beta}} \end{bmatrix}$, where

$$\frac{\partial \boldsymbol{\psi}_{\boldsymbol{\alpha}}}{\partial \boldsymbol{\alpha}} = -\sum_{i=n_1+1}^{n_1+n_2} \mathbf{d}_i \mathbf{d}_i^T \quad \text{and} \quad \frac{\partial \boldsymbol{\psi}_{\boldsymbol{\alpha}}}{\partial \boldsymbol{\beta}} = \mathbf{0}.$$

In an external validation study,

$$\frac{\partial \boldsymbol{\psi}_{\boldsymbol{\beta}}}{\partial \boldsymbol{\beta}} = -\sum_{i=1}^{n_1} \widehat{\mathbf{X}}_i^T \boldsymbol{\Sigma}_i^{-1} \widehat{\mathbf{X}}_i,$$

and

$$\frac{\partial \boldsymbol{\psi}_{\boldsymbol{\beta}}}{\partial \alpha_l} = \sum_{i=1}^{n_1} \left[ \frac{\partial \widehat{\mathbf{X}}_i^T}{\partial \alpha_l} \boldsymbol{\Sigma}_i^{-1} (\mathbf{Y}_i - \widehat{\mathbf{X}}_i \boldsymbol{\beta}) - \widehat{\mathbf{X}}_i^T \boldsymbol{\Sigma}_i^{-1} \frac{\partial \widehat{\mathbf{X}}_i}{\partial \alpha_l} \boldsymbol{\beta} \right],$$

where

$$\frac{\partial \widehat{\mathbf{X}}_i}{\partial \alpha_l} = \begin{bmatrix} 0 & \frac{\partial \widehat{X}_i(t_{i1})}{\partial \alpha_l} & 0 & \frac{\partial \widehat{X}_i(t_{i1})}{\partial \alpha_l} t_{i1} & 0 \\ 0 & \frac{\partial \widehat{X}_i(t_{i2})}{\partial \alpha_l} & 0 & \frac{\partial \widehat{X}_i(t_{i2})}{\partial \alpha_l} t_{i2} & 0 \\ \vdots & & & & \\ 0 & \frac{\partial \widehat{X}_i(t_{im_i})}{\partial \alpha_l} & 0 & \frac{\partial \widehat{X}_i(t_{im_i})}{\partial \alpha_l} t_{im_i} & 0 \end{bmatrix}, \quad \text{for } l = 0, 1, 2, 3, 4.$$

Based on Equation (5) and (6) in the main paper, we have

$$\frac{\partial \widehat{X_i}(t_{ij})}{\partial \alpha_0} = 1, \quad j = 1, 2, ...m_i;$$

$$\frac{\partial \widehat{X_i}(t_{ij})}{\partial \alpha_1} = Z_i(t_{ij}), \quad j = 1, 2, ...m_i;$$

$$\frac{\partial \widehat{X_i}(t_{i1})}{\partial \alpha_2} = t_{i1}; \qquad \frac{\partial \widehat{X_i}(t_{ij})}{\partial \alpha_2} = \frac{\sum_{k=1}^{j-1} t_{ik}(t_{i(k+1)} - t_{ik})}{t_{ij} - t_{i1}}, \quad j = 2, ...m_i;$$

$$\frac{\partial \widehat{X_i}(t_{i1})}{\partial \alpha_3} = C_i(t_{i1})t_{i1}; \qquad \frac{\partial \widehat{X_i}(t_{ij})}{\partial \alpha_3} = \frac{\sum_{k=1}^{j-1} C_i(t_{ik})t_{ik}(t_{i(k+1)} - t_{ik})}{t_{ij} - t_{i1}}, \quad j = 2, ...m_i;$$

$$\frac{\partial \widehat{X_i}(t_{i1})}{\partial \alpha_4} = W_i(t_{i1}); \qquad \frac{\partial \widehat{X_i}(t_{ij})}{\partial \alpha_4} = \frac{\sum_{k=1}^{j-1} W_i(t_{ik})(t_{i(k+1)} - t_{ik})}{t_{ij} - t_{i1}}, \quad j = 2, ...m_i.$$

With an internal validation study, replace $n_1$ in $\frac{\partial \boldsymbol{\psi}_{\boldsymbol{\beta}}}{\partial \boldsymbol{\beta}}$ and $\frac{\partial \boldsymbol{\psi}_{\boldsymbol{\beta}}}{\partial \alpha_l}$ with $n_1 + n_2$.

## A.2 For Validation Studies With Repeated Measures

Assume that for the $i$th participant, $\mathbf{A}_i = (\mathbf{d}_i(t_{i1}), ..., \mathbf{d}_i(t_{il_i}))^T$, where $l_i$ is the number repeated measurements in the validation study; $\mathbf{B}_i$ is the working correlation matrix. The changes to $\mathbf{A}(\boldsymbol{\theta})$ and $\mathbf{B}(\boldsymbol{\theta})$ defined in Appendix A.1 are

$$\text{Var}(\boldsymbol{\psi}_{\boldsymbol{\alpha}}) = \sum_{i=n_1+1}^{n_1+n_2} \mathbf{A}_i^T \mathbf{B}_i^{-1} (\mathbf{c}_i - \mathbf{A}_i \boldsymbol{\alpha})(\mathbf{c}_i - \mathbf{A}_i \boldsymbol{\alpha})^T \mathbf{B}_i^{-1} \mathbf{A}_i,$$

$$\frac{\partial \boldsymbol{\psi}_{\boldsymbol{\alpha}}}{\partial \boldsymbol{\alpha}} = -\sum_{i=n_1+1}^{n_1+n_2} \mathbf{A}_i^T \mathbf{B}_i^{-1} \mathbf{A}_i.$$

## A.3 Consistency of the Estimators

We prove the consistency of the estimators by showing the mean of each estimating function $\boldsymbol{\psi}_{\boldsymbol{\alpha}}, \boldsymbol{\psi}_{\boldsymbol{\beta}}$ is zero. Then, under standard regularity conditions, GEE theory establishes consistency.

For $\boldsymbol{\psi}_{\boldsymbol{\alpha}}$: since $\boldsymbol{\psi}_{\boldsymbol{\alpha}} = \sum_{i=n_1+1}^{n_1+n_2} \mathbf{d}_i(c_i - \mathbf{d}_i^T \boldsymbol{\alpha})$,

$$\begin{aligned}
\mathbb{E}[\boldsymbol{\psi}_{\boldsymbol{\alpha}}] &= \mathbb{E}[\sum_{i=n_1+1}^{n_1+n_2} \mathbf{d}_i(c_i - \mathbf{d}_i^T \boldsymbol{\alpha})] \\
&= \sum_{i=n_1+1}^{n_1+n_2} \mathbf{d}_i(\mathbb{E}[c_i] - \mathbf{d}_i^T \boldsymbol{\alpha}) \\
&= 0.
\end{aligned}$$

For $\boldsymbol{\psi}_{\boldsymbol{\beta}}$: since $\boldsymbol{\psi}_{\boldsymbol{\beta}} = \sum_{i=1}^{N} \widehat{\mathbf{X}}_i^T \boldsymbol{\Sigma}_i^{-1}(\mathbf{Y}_i - \widehat{\mathbf{X}}_i \boldsymbol{\beta})$,

$$\begin{aligned}
\mathbb{E}[\boldsymbol{\psi}_{\boldsymbol{\beta}}] &= \mathbb{E}_{\mathbf{Y}|\widetilde{\mathbf{C}},\widetilde{\mathbf{t}},\widetilde{\mathbf{W}}}[\sum_{i=1}^{N} \widehat{\mathbf{X}}_i^T \boldsymbol{\Sigma}_i^{-1}(\mathbf{Y}_i - \widehat{\mathbf{X}}_i \boldsymbol{\beta})] \\
&= \sum_{i=1}^{N} \widehat{\mathbf{X}}_i^T \boldsymbol{\Sigma}_i^{-1}[(\mathbb{E}_{\mathbf{Y}_i|\widetilde{\mathbf{C}}_i,\widetilde{\mathbf{t}}_i,\widetilde{\mathbf{W}}_i}[\mathbf{Y}_i]) - \widehat{\mathbf{X}}_i \boldsymbol{\beta}] \\
&= 0, \text{based on equation (3) in the main paper.}
\end{aligned}$$

## A.4 Equivalence of the Two-Step Approach to a Simultaneous Solution to Joint Estimating Equations

It is noteworthy that solving the system of estimating equation $\boldsymbol{\psi} = \mathbf{0}$ (Section 2.3 Estimation) to obtain $\widehat{\boldsymbol{\alpha}}$ and $\widehat{\boldsymbol{\beta}}$ is equivalent to performing the following two steps: First, we estimate $\boldsymbol{\alpha}$ in the VS. Then, following equation (5) in the main paper, the expectation of the true exposure history function, $\mathbb{E}[X_i(t_{ij})|\widetilde{C}_i(t_{ij}), \widetilde{t}_{ij}, \widetilde{\mathbf{W}}_i(t_{ij})]$, can be estimated as

$$\widehat{\mathbb{E}}[X_i(t_{ij})|\widetilde{C}_i(t_{ij}), \widetilde{t}_{ij}, \widetilde{\mathbf{W}}_i(t_{ij})] = \frac{\sum_{k=1}^{j-1}(t_{i(k+1)} - t_{ik}) f(C_i(t_{ik}), t_{ik}, \mathbf{W}_i(t_{ik}); \widehat{\boldsymbol{\alpha}})}{t_{ij} - t_{i1}}.$$

Next, with outcome variable, $Y_i(t_{ij})$, and explanatory variables $\widehat{\mathbb{E}}[X_i(t_{ij})|\widetilde{C}_i(t_{ij}), \widetilde{t}_{ij}, \widetilde{\mathbf{W}}_i(t_{ij})]$, $t_{ij}$, $\mathbf{W}_i(t_{ij})$, GEE can be applied to obtain estimates for $\boldsymbol{\beta}$.

The proof of the equivalence is as follows. Firstly, assume $\widehat{\boldsymbol{\alpha}}, \widehat{\boldsymbol{\beta}}$ are estimates of the two-step approach, as proposed in this paper. From the first step, $\boldsymbol{\psi}_{\boldsymbol{\alpha}}(\widehat{\boldsymbol{\alpha}}) = 0$. From the second step, $\widehat{\boldsymbol{\beta}}$ is the root to $\boldsymbol{\psi}_{\boldsymbol{\beta}}(\boldsymbol{\beta}; \widehat{\boldsymbol{\alpha}})$; that is, $\boldsymbol{\psi}_{\boldsymbol{\beta}}(\widehat{\boldsymbol{\alpha}}, \widehat{\boldsymbol{\beta}}) = 0$. Therefore, $\widehat{\boldsymbol{\alpha}}, \widehat{\boldsymbol{\beta}}$ satisfy both equations (i.e., the joint equations $\boldsymbol{\psi}_{\boldsymbol{\alpha}}(\boldsymbol{\alpha}) = 0$ and $\boldsymbol{\psi}_{\boldsymbol{\beta}}(\boldsymbol{\beta}; \boldsymbol{\alpha}) = 0$).

Secondly, assume $\widehat{\boldsymbol{\alpha}}^*, \widehat{\boldsymbol{\beta}}^*$ are the solutions to the joint equations. Then, $\widehat{\boldsymbol{\alpha}}^*$ is the estimate of the first step (due to the fact that it is the solution to the estimating equation $\boldsymbol{\psi}_{\boldsymbol{\alpha}}(\boldsymbol{\alpha}) = 0$). Since $\boldsymbol{\psi}_{\boldsymbol{\beta}}(\widehat{\boldsymbol{\alpha}}^*, \widehat{\boldsymbol{\beta}}^*) = 0$, $\widehat{\boldsymbol{\beta}}^*$ is the estimate from the second step. Therefore, $\widehat{\boldsymbol{\alpha}}^*, \widehat{\boldsymbol{\beta}}^*$ are estimates from the two-step approach.

Thus, the two-step approach is equivalent to a simultaneous solution to joint estimating equations.

## A.5 Considerations of the Time Scale for Analysis

In this section, we show that the time scale used in the measurement error model does not have to be the same as that used in the main model for valid application of the methods described in this paper. For subject $i$ in the $j$th occasion, let $t_{ij}$ and $t'_{ij}$ denote time since baseline cognitive assessment and age, respectively. When time since baseline is used as the time scale in the outcome model and we want to use age as the time scale in the measurement error model, baseline age, $t'_{i1}$, should be considered in the outcome model as a potential confounder. Let $\mathbf{W}_i(t_{ij})_{-t'_{i1}}$ denote all potential confounders excluding $t'_{i1}$. We can rewrite the potential confounders $\widetilde{\mathbf{W}}_i(t_{ij})$ as $(t'_{i1}, \widetilde{\mathbf{W}}_i(t_{ij})_{-t'_{i1}})$. It follows that formula (3) in the main paper can be rewritten as

$$\begin{aligned}\mathbb{E}[Y_i(t_{ij})|\ \widetilde{C}_i(t_{ij}), \widetilde{t}_{ij}, \widetilde{\mathbf{W}}_i(t_{ij})] =& \beta_0 + \beta_1 \mathbb{E}[X_i(t_{ij})|\widetilde{C}_i(t_{ij}), \widetilde{t}_{ij}, t'_{i1}, \widetilde{\mathbf{W}}_i(t_{ij})_{-t'_{i1}}] + \beta_2 t_{ij} \\ &+ \beta_3 t_{ij} \mathbb{E}[X_i(t_{ij})|\widetilde{C}_i(t_{ij}), \widetilde{t}_{ij}, t'_{i1}, \widetilde{\mathbf{W}}_i(t_{ij})_{-t'_{i1}}] + \boldsymbol{\beta}_4^T \mathbf{W}_i(t_{ij}).\end{aligned}$$

Following formula (4) in the main paper,

$$\begin{aligned}&\mathbb{E}[X_i(t_{ij})|\widetilde{C}_i(t_{ij}), \widetilde{t}_{ij}, t'_{i1}, \widetilde{\mathbf{W}}_i(t_{ij})_{-t'_{i1}}] \\ &= \frac{\sum_{k=1}^{j-1}(t_{i(k+1)} - t_{ik})\mathbb{E}[c_i(t_{ik})|\ C_i(t_{i1}), ..., C_i(t_{ij}), t_{i2}, ..., t_{ij}, t'_{i1}, \mathbf{W}_i(t_{i1})_{-t'_{i1}}, ..., \mathbf{W}_i(t_{ij})_{-t'_{i1}}]}{t_{ij} - t_{i1}}.\end{aligned}$$

Note that $t_{i1}$ is not included as it has a fixed value 0.

Since age at the $k$th occasion, $t'_{ik}$, can be written as the sum of age at baseline and time from baseline to the $k$th occasion (i.e., $t'_{ik} = t_{ik} + t'_{i1}$), given the baseline age $t'_{i1}$, there is a one-to-one transformation between $t_{ik}$ and $t'_{ik}$. It follows that, given the baseline age, the surrogate

exposure, $C_i(t_{ik})$, for the $i$th subject at the $k$th occasion, which uses time since baseline, $t_{ik}$, as the time index, can also be written as $C_i(t'_{ik})$, which uses age as the time index, $k = 1, \ldots, j$. Similarly, $c_i(t_{ik})$ and $\mathbf{W}_i(t_{ik})$ can also be written as $c_i(t'_{ik})$ and $\mathbf{W}_i(t'_{ik})$, respectively. Therefore, $\mathbb{E}[c_i(t_{ik})|C_i(t_{i1}), ..., C_i(t_{ij}), t_{i2}, ..., t_{ij}, t'_{i1}, \mathbf{W}_i(t_{i1})_{-t'_{i1}}, ..., \mathbf{W}_i(t_{ij})_{-t'_{i1}}] = \mathbb{E}[c_i(t'_{ik})|C_i(t'_{i1}), ..., C_i(t'_{ij}), t'_{i1}, ..., t'_{ij}, \mathbf{W}_i(t'_{i1})_{-t'_{i1}}, ..., \mathbf{W}_i(t'_{ij})_{-t'_{i1}}]$.

Furthermore, assuming the following localized error assumption with age as the time scale

$$\begin{aligned}&\mathbb{E}[c_i(t'_{ik})|C_i(t'_{i1}), ..., C_i(t'_{ij}), t'_{i1}, ..., t'_{ij}, \mathbf{W}_i(t'_{i1})_{-t'_{i1}}, ..., \mathbf{W}_i(t'_{ij})_{-t'_{i1}}]\\ =&\mathbb{E}[c_i(t'_{ik})|C_i(t'_{ik}), t'_{ik}, \mathbf{W}_i(t'_{ik})_{-t'_{i1}}],\end{aligned}$$

formula (3) can be written as

$$\begin{aligned}\mathbb{E}[Y_i(t_{ij})|\widetilde{C}_i(t_{ij}), \widetilde{t}_{ij}, \widetilde{\mathbf{W}}_i(t_{ij})] = \beta_0 + \beta_1 &\frac{\sum_{k=1}^{j-1}(t_{i(k+1)} - t_{ik})\mathbb{E}[c_i(t'_{ik})|C_i(t'_{ik}), t'_{ik}, \mathbf{W}_i(t'_{ik})_{-t'_{i1}}]}{t_{ij} - t_{i1}} + \beta_2 t_{ij}\\ + \beta_3 t_{ij} &\frac{\sum_{k=1}^{j-1}(t_{i(k+1)} - t_{ik})\mathbb{E}[c_i(t'_{ik})|C_i(t'_{ik}), t'_{ik}, \mathbf{W}_i(t'_{ik})_{-t'_{i1}}]}{t_{ij} - t_{i1}} + \boldsymbol{\beta}_4^T \mathbf{W}_i(t_{ij}),\end{aligned}$$

where the time scale for the outcome model is time since baseline, while it is age in the measurement error model. This proves that we can use the two different time scales in the outcome and measurement error models under the above localized error assumption, which states that conditional on all the variables at the current age, the true exposure is independent of these variables in the past.

It is easy to show that the above derivation still holds if the interactions between $t_{ij}$ and some or all elements of $\mathbf{W}_i(t_{ij})$ are also included in the outcome model. In addition, it holds if we replace age by calendar time, in which case $t'_{i1}$ is the calendar time at baseline and $\mathbf{W}_i(t_{ij})$ may include age at $t_{ij}$.

## A.6 Relationship between Classical-Type Measurement Error Model and Our Assumed Measurement Error Model

The classical-type error model can be viewed as a special case of our assumed model (see Chapter 2 of Carroll et al., 2006 for more discussion). For example, the classical additive measurement error model $C_{ij} = c_i + e_{ij}$, with assumptions $e_{ij} \sim (0, \sigma^2)$, $c_i \sim (\mu_c, \sigma_c^2)$, $\mathrm{Cov}(c_i, e_{ij}) = 0$, where $C_{ij}$ is the surrogate exposure for the $j$th occasion of the $i$th individual, $c_i$ is the true exposure of the $i$th individual and $e_{ij}$ is corresponding error, implies that $\mathbb{E}(c_i|C_{ij}) = \gamma_0 + \gamma_1 C_{ij}$, where $\gamma_0 = \mu_c \frac{\sigma^2}{\sigma_c^2 + \sigma^2}$, $\gamma_1 = \frac{\sigma_c^2}{\sigma_c^2 + \sigma^2}$.

# Web Appendix B Simulation Study

## B.1 Data Generation Process

For each individual $i$, we generated the first observed time point $t_{i1}$ from a uniform distribution with the minimum value 0 and the maximum value 10. Then $t_{ij} = t_{i1} + (j-1),\ j = 2, 3, 4, 5$, to create a 10-year variation in study entry time, each individual was followed for 5 years, and information was gathered once every year.

When there were no additional covariate in the outcome model, we generated the surrogate exposure $\mathbf{C}_i = (C_i(t_{i1}), ...C_i(t_{i5}))^T$ from MVN$(\mathbf{0}, \Sigma_C)$, where $\Sigma_C$ followed a first-order autoregressive correlation matrix (AR(1)) structure with variance $\sigma_C^2 = 1$ and the correlation for adjacent elements $\rho_C = 0.6$. When a covariate was to be considered, we generated the surrogate exposure $\mathbf{C}$ and $\mathbf{W}$ together, i.e.,

$$(C_i(t_{i1}), ...C_i(t_{i5}), W_i(t_{i1}), ...W_i(t_{i5}))^T \sim \text{MVN}(\mathbf{0}, \Sigma)$$

$$\Sigma = \begin{bmatrix} \Sigma_C & \Sigma_{C,W} \\ \Sigma_{W,C} & \Sigma_W \end{bmatrix},$$

where $\Sigma_C$ followed an AR(1) structure with variance $\sigma_C^2 = 1$ and correlation parameter $\rho_C = 0.6$, $\Sigma_W$ followed an AR(1) structure with variance $\sigma_W^2 = 1$ and correlation parameter $\rho_W = 0.2$, $\Sigma_{C,W} = \Sigma_{W,C}$ followed a correlation structure with the diagonal element $\rho_{C,W} = 0.4$, representing an intermediate level of correlation, when $(C, W)$ were assumed correlated and $\rho_{C,W} = 0$ when $(C, W)$ were assumed uncorrelated.

Next, to generate the true exposure $c$ from the surrogate exposure $C$, we needed to specify the parameters in the MEM. When included in the model, $\alpha_0 = 1.2, \alpha_1 = 0.7, \alpha_2 = 0.6, \alpha_3 = 0.5, \alpha_4 = 0.4$. These choices were motivated by the correlation between $c$ and $C$ (0.61) in the illustrative example, resulting in Corr$(c, C)$ ranging from 0.57 to 0.85, depending upon the exact model used to generate the data.

## B.2 Additional Results

Below is a summary of all simulation tables for this paper. Scenarios under True MEM are as defined in Section 3 in the main manuscript. We only considered AR(1) as the working correlation in scenarios where $W$ was included; because when we applied our method for scenarios where $W$ was not present, we found the bias and coverage probability were invariant to the choice of the correlation structure. Note that Tables Main 1-Main 4 can be found in the main paper, and Tables Supplement 1-12 can be found below, with page numbers provided in this table.

| | | Working Correlation for the Outcome Model | | |
|---|---|---|---|---|
| | | AR(1) | Unstructured | Independence |
| Validation Study | True MEM | | | |
| EVS | NI-NW | Main 1 | Supp 5; p12 | Supp 7; p14 |
| | NI-WP; NI-WC | Main 2 | — | — |
| | IP-NW | Main 3 | Supp 6; p13 | Supp 8; p15 |
| | IP-WP; IP-WC | Main 4 | — | — |
| IVS | NI-NW | Supp 1; p8 | Supp 9; p16 | Supp 11; p18 |
| | NI-WP; NI-WC | Supp 2; p9 | — | — |
| | IP-NW | Supp 3; p10 | Supp 10; p17 | Supp 12; p19 |
| | IP-WP; IP-WC | Supp 4; p11 | — | — |

Supplementary Table 1: Bias (%), empirical standard error (ESE), estimated sandwich standard error (SE), and coverage probability (CP) of 95% confidence interval for the interaction effect estimate $\widehat{\beta}_3$ based on 1000 simulations, under the IVS Scenario NI-NW in the true MEM. Working correlation specified as AR(1) in the GEE analysis for the outcome model.

| | | | Fitting Method | |
|---|---|---|---|---|
| | | Sample Size | Naive Method | Proposed Method |
| $\beta_1$ | $\beta_3$ | $n_1$, $n_2$ | Bias (%) (ESE) CP | Bias (%) (ESE) (SE) CP |
| 3 | 3 | 1000, 100 | -30.06 (0.10) 0.00 | 1.89 (0.45) (0.44) 0.95 |
| | | 500 | -29.87 (0.08) 0.00 | 0.49 (0.21) (0.20) 0.94 |
| | | 5000, 100 | -30.05 (0.05) 0.00 | 2.28 (0.45) (0.43) 0.93 |
| | | 500 | -30.06 (0.04) 0.00 | 0.24 (0.18) (0.19) 0.95 |
| 3 | 0.2 | 1000, 100 | -29.97 (0.02) 0.16 | 2.13 (0.04) (0.04) 0.95 |
| | | 500 | -29.49 (0.02) 0.07 | 1.04 (0.03) (0.03) 0.93 |
| | | 5000, 100 | -30.15 (0.01) 0.00 | 2.13 (0.03) (0.03) 0.94 |
| | | 500 | -30.29 (0.01) 0.00 | -0.10 (0.02) (0.02) 0.96 |
| 0.2 | 3 | 1000, 100 | -30.06 (0.09) 0.00 | 1.89 (0.44) (0.43) 0.95 |
| | | 500 | -29.89 (0.08) 0.00 | 0.46 (0.20) (0.19) 0.94 |
| | | 5000, 100 | -30.03 (0.04) 0.00 | 2.30 (0.45) (0.42) 0.93 |
| | | 500 | -30.05 (0.04) 0.00 | 0.25 (0.18) (0.19) 0.95 |
| 0.2 | 0.2 | 1000, 100 | -30.04 (0.01) 0.00 | 1.94 (0.03) (0.03) 0.95 |
| | | 500 | -29.81 (0.01) 0.00 | 0.56 (0.02) (0.02) 0.95 |
| | | 5000, 100 | -30.00 (0.00) 0.00 | 2.35 (0.03) (0.03) 0.94 |
| | | 500 | -30.14 (0.00) 0.00 | 0.12 (0.01) (0.01) 0.95 |

Supplementary Table 2: Bias (%), empirical standard error (ESE), estimated sandwich standard error (SE), and coverage probability (CP) of 95% confidence interval for the interaction effect estimate $\widehat{\beta}_3$ based on 1000 simulations, under the IVS Scenario NI-WP and NI-WC in the true MEM. Working correlation specified as AR(1) in the GEE analysis for the outcome model.

| | | | Fitting Method | | |
|---|---|---|---|---|---|
| | | Sample Size | Naive Method | Proposed, with W | Proposed, no W |
| $\beta_1$ | $\beta_3$ | $n_1$, $n_2$ | Bias (%) (ESE) CP | Bias (%) (ESE) (SE) CP | Bias (%) (ESE) (SE) CP |
| Results for NI-WP | | | | | |
| 3 | 3 | 1000, 100 | -30.04 (0.11) 0.00 | -0.15 (0.37) (0.37) 0.93 | 2.08 (0.49) (0.48) 0.94 |
| | | 500 | -29.96 (0.09) 0.00 | -0.01 (0.18) (0.18) 0.94 | 0.24 (0.23) (0.22) 0.95 |
| | | 5000, 100 | -29.93 (0.05) 0.00 | 0.99 (0.39) (0.36) 0.94 | 3.01 (0.53) (0.47) 0.93 |
| | | 500 | -30.03 (0.05) 0.00 | 0.32 (0.16) (0.16) 0.95 | 0.78 (0.20) (0.21) 0.95 |
| 3 | 0.2 | 1000, 100 | -30.02 (0.02) 0.19 | 0.12 (0.03) (0.04) 0.95 | 2.08 (0.04) (0.04) 0.95 |
| | | 500 | -30.14 (0.02) 0.10 | -0.02 (0.02) (0.02) 0.95 | 0.01 (0.03) (0.03) 0.95 |
| | | 5000, 100 | -29.88 (0.01) 0.00 | 1.34 (0.03) (0.03) 0.94 | 3.05 (0.04) (0.03) 0.94 |
| | | 500 | -30.15 (0.01) 0.00 | 0.20 (0.02) (0.02) 0.96 | 0.60 (0.02) (0.02) 0.95 |
| 0.2 | 3 | 1000, 100 | -30.04 (0.10) 0.00 | -0.18 (0.36) (0.36) 0.93 | 2.07 (0.49) (0.47) 0.94 |
| | | 500 | -29.96 (0.08) 0.00 | -0.01 (0.18) (0.17) 0.94 | 0.25 (0.22) (0.21) 0.95 |
| | | 5000, 100 | -29.94 (0.04) 0.00 | 0.97 (0.39) (0.36) 0.94 | 3.01 (0.53) (0.47) 0.93 |
| | | 500 | -30.02 (0.04) 0.00 | 0.33 (0.16) (0.16) 0.95 | 0.79 (0.20) (0.20) 0.95 |
| 0.2 | 0.2 | 1000, 100 | -30.12 (0.01) 0.00 | -0.28 (0.03) (0.03) 0.94 | 1.94 (0.03) (0.03) 0.94 |
| | | 500 | -30.05 (0.01) 0.00 | -0.07 (0.01) (0.01) 0.94 | 0.12 (0.02) (0.02) 0.94 |
| | | 5000, 100 | -29.90 (0.00) 0.00 | 1.11 (0.03) (0.02) 0.94 | 3.05 (0.04) (0.03) 0.93 |
| | | 500 | -30.07 (0.00) 0.00 | 0.32 (0.01) (0.01) 0.95 | 0.72 (0.01) (0.01) 0.95 |
| Results for NI-WC | | | | | |
| 3 | 3 | 1000, 100 | -17.47 (0.11) 0.00 | -0.26 (0.32) (0.32) 0.94 | -2.59 (0.37) (0.36) 0.90 |
| | | 500 | -17.45 (0.09) 0.00 | -0.14 (0.16) (0.15) 0.94 | -3.91 (0.18) (0.17) 0.85 |
| | | 5000, 100 | -17.38 (0.05) 0.00 | 0.55 (0.33) (0.31) 0.93 | -2.13 (0.39) (0.35) 0.89 |
| | | 500 | -17.50 (0.05) 0.00 | 0.21 (0.14) (0.14) 0.95 | -3.47 (0.16) (0.16) 0.87 |
| 3 | 0.2 | 1000, 100 | -25.69 (0.02) 0.34 | -0.40 (0.03) (0.03) 0.95 | -12.32 (0.03) (0.03) 0.81 |
| | | 500 | -25.94 (0.02) 0.20 | -0.29 (0.02) (0.02) 0.95 | -13.80 (0.02) (0.02) 0.76 |
| | | 5000, 100 | -25.58 (0.01) 0.00 | 0.40 (0.02) (0.02) 0.94 | -11.87 (0.03) (0.02) 0.72 |
| | | 500 | -25.93 (0.01) 0.00 | 0.04 (0.01) (0.01) 0.95 | -13.33 (0.01) (0.01) 0.53 |
| 0.2 | 3 | 1000, 100 | -16.96 (0.10) 0.00 | -0.26 (0.31) (0.32) 0.94 | -1.99 (0.37) (0.36) 0.91 |
| | | 500 | -16.92 (0.08) 0.00 | -0.13 (0.15) (0.15) 0.94 | -3.30 (0.17) (0.16) 0.87 |
| | | 5000, 100 | -16.87 (0.04) 0.00 | 0.56 (0.33) (0.31) 0.93 | -1.52 (0.39) (0.35) 0.89 |
| | | 500 | -16.98 (0.04) 0.00 | 0.22 (0.14) (0.14) 0.95 | -2.86 (0.16) (0.16) 0.89 |
| 0.2 | 0.2 | 1000, 100 | -19.54 (0.01) 0.02 | -0.40 (0.02) (0.02) 0.93 | -5.05 (0.03) (0.03) 0.87 |
| | | 500 | -19.50 (0.01) 0.00 | -0.20 (0.01) (0.01) 0.94 | -6.30 (0.01) (0.01) 0.79 |
| | | 5000, 100 | -19.32 (0.00) 0.00 | 0.55 (0.02) (0.02) 0.94 | -4.44 (0.03) (0.02) 0.85 |
| | | 500 | -19.52 (0.00) 0.00 | 0.17 (0.01) (0.01) 0.95 | -5.83 (0.01) (0.01) 0.77 |

Supplementary Table 3: Bias (%), empirical standard error (ESE), estimated sandwich standard error (SE), and coverage probability (CP) of 95% confidence interval for the interaction effect estimate $\widehat{\beta}_3$ based on 1000 simulations, under the IVS Scenario IP-NW in the true MEM. Working correlation specified as AR(1) in the GEE analysis for the outcome model.

| | | | Fitting Method | | |
|---|---|---|---|---|---|
| | | Sample Size | Naive Method | Proposed, with Interaction | Proposed, no Interaction |
| $\beta_1$ | $\beta_3$ | $n_1$, $n_2$ | Bias (%) (ESE) CP | Bias (%) (ESE) (SE) CP | Bias (%) (ESE) (SE) CP |
| 3 | 3 | 1000, 100 | 545.52 (0.32) 0.00 | -0.15 (0.10) (0.10) 0.92 | 42.82 (0.27) (0.25) 0.00 |
| | | 500 | 545.77 (0.27) 0.00 | -0.01 (0.05) (0.05) 0.93 | 43.00 (0.12) (0.11) 0.00 |
| | | 5000, 100 | 545.55 (0.15) 0.00 | 0.04 (0.10) (0.10) 0.93 | 43.41 (0.26) (0.25) 0.00 |
| | | 500 | 545.55 (0.15) 0.00 | -0.03 (0.04) (0.05) 0.95 | 43.00 (0.12) (0.12) 0.00 |
| 3 | 0.2 | 1000, 100 | 1067.41 (0.04) 0.00 | -0.22 (0.02) (0.02) 0.93 | 144.53 (0.03) (0.03) 0.00 |
| | | 500 | 1067.59 (0.03) 0.00 | -0.05 (0.01) (0.01) 0.93 | 144.96 (0.01) (0.01) 0.00 |
| | | 5000, 100 | 1067.42 (0.02) 0.00 | 0.34 (0.02) (0.02) 0.92 | 145.74 (0.03) (0.03) 0.00 |
| | | 500 | 1067.50 (0.02) 0.00 | -0.10 (0.01) (0.01) 0.94 | 145.04 (0.01) (0.01) 0.00 |
| 0.2 | 3 | 1000, 100 | 510.72 (0.32) 0.00 | -0.15 (0.09) (0.09) 0.93 | 36.16 (0.26) (0.24) 0.00 |
| | | 500 | 510.98 (0.27) 0.00 | -0.01 (0.04) (0.04) 0.93 | 36.32 (0.11) (0.10) 0.00 |
| | | 5000, 100 | 510.76 (0.15) 0.00 | 0.02 (0.09) (0.09) 0.93 | 36.71 (0.25) (0.24) 0.00 |
| | | 500 | 510.75 (0.14) 0.00 | -0.03 (0.04) (0.04) 0.95 | 36.31 (0.11) (0.11) 0.00 |
| 0.2 | 0.2 | 1000, 100 | 554.85 (0.02) 0.00 | -0.15 (0.01) (0.01) 0.94 | 45.08 (0.02) (0.02) 0.00 |
| | | 500 | 555.15 (0.02) 0.00 | 0.00 (0.00) (0.00) 0.94 | 45.29 (0.01) (0.01) 0.00 |
| | | 5000, 100 | 554.96 (0.01) 0.00 | 0.06 (0.01) (0.01) 0.93 | 45.72 (0.02) (0.02) 0.00 |
| | | 500 | 554.89 (0.01) 0.00 | -0.04 (0.00) (0.00) 0.95 | 45.29 (0.01) (0.01) 0.00 |

Supplementary Table 4: Bias (%), empirical standard error (ESE), estimated sandwich standard error (SE), and coverage probability (CP) of 95% confidence interval for the interaction effect estimate $\widehat{\beta}_3$ based on 1000 simulations, under the IVS Scenario IP-WP and IP-WC in the true MEM. Working correlation specified as AR(1) in the GEE analysis for the outcome model.

| | | | Fitting Method | | |
|---|---|---|---|---|---|
| | | Sample Size | Naive Method | Proposed, with W | Proposed, no W |
| $\beta_1$ | $\beta_3$ | $n_1$, $n_2$ | Bias (%) (ESE) | Bias (%) (ESE) (SE) CP | Bias (%) (ESE) (SE) CP |
| Results for IP-WU | | | | | |
| 3 | 3 | 1000, 100 | 544.32 (0.32) 0.00 | -0.19 (0.11) (0.10) 0.93 | -0.30 (0.12) (0.11) 0.93 |
| | | 500 | 544.81 (0.28) 0.00 | -0.06 (0.05) (0.05) 0.94 | -0.06 (0.05) (0.05) 0.94 |
| | | 5000, 100 | 544.60 (0.15) 0.00 | 0.07 (0.10) (0.10) 0.93 | 0.03 (0.12) (0.11) 0.91 |
| | | 500 | 544.66 (0.14) 0.00 | -0.01 (0.05) (0.04) 0.94 | -0.02 (0.05) (0.05) 0.94 |
| 3 | 0.2 | 1000, 100 | 1063.75 (0.04) 0.00 | -0.10 (0.02) (0.02) 0.93 | -0.53 (0.02) (0.02) 0.92 |
| | | 500 | 1064.25 (0.03) 0.00 | -0.10 (0.01) (0.01) 0.95 | -0.09 (0.01) (0.01) 0.95 |
| | | 5000, 100 | 1064.12 (0.02) 0.00 | 0.38 (0.02) (0.02) 0.92 | 0.24 (0.02) (0.02) 0.92 |
| | | 500 | 1064.07 (0.02) 0.00 | -0.10 (0.01) (0.01) 0.93 | -0.15 (0.01) (0.01) 0.94 |
| 0.2 | 3 | 1000, 100 | 509.71 (0.32) 0.00 | -0.19 (0.10) (0.09) 0.93 | -0.29 (0.11) (0.10) 0.93 |
| | | 500 | 510.20 (0.27) 0.00 | -0.06 (0.04) (0.04) 0.95 | -0.06 (0.05) (0.04) 0.94 |
| | | 5000, 100 | 509.99 (0.15) 0.00 | 0.04 (0.09) (0.09) 0.92 | 0.02 (0.11) (0.10) 0.91 |
| | | 500 | 510.05 (0.14) 0.00 | -0.01 (0.04) (0.04) 0.94 | -0.01 (0.05) (0.04) 0.94 |
| 0.2 | 0.2 | 1000, 100 | 553.19 (0.02) 0.00 | -0.23 (0.01) (0.01) 0.93 | -0.35 (0.01) (0.01) 0.92 |
| | | 500 | 553.67 (0.02) 0.00 | -0.08 (0.00) (0.00) 0.94 | -0.07 (0.00) (0.00) 0.94 |
| | | 5000, 100 | 553.58 (0.01) 0.00 | 0.06 (0.01) (0.01) 0.92 | 0.02 (0.01) (0.01) 0.92 |
| | | 500 | 553.58 (0.01) 0.00 | -0.02 (0.00) (0.00) 0.94 | -0.03 (0.00) (0.00) 0.94 |
| Results for IP-WC | | | | | |
| 3 | 3 | 1000, 100 | 557.14 (0.32) 0.00 | -0.21 (0.11) (0.10) 0.92 | -1.80 (0.11) (0.11) 0.89 |
| | | 500 | 557.57 (0.27) 0.00 | -0.06 (0.05) (0.04) 0.94 | -1.60 (0.05) (0.05) 0.81 |
| | | 5000, 100 | 557.40 (0.15) 0.00 | 0.02 (0.10) (0.10) 0.93 | -1.49 (0.11) (0.10) 0.88 |
| | | 500 | 557.44 (0.14) 0.00 | -0.02 (0.05) (0.04) 0.94 | -1.57 (0.05) (0.05) 0.82 |
| 3 | 0.2 | 1000, 100 | 1067.13 (0.04) 0.00 | -0.21 (0.02) (0.02) 0.93 | -4.72 (0.02) (0.02) 0.89 |
| | | 500 | 1067.46 (0.03) 0.00 | -0.11 (0.01) (0.01) 0.94 | -4.30 (0.01) (0.01) 0.83 |
| | | 5000, 100 | 1067.37 (0.02) 0.00 | 0.24 (0.02) (0.02) 0.93 | -3.97 (0.02) (0.02) 0.89 |
| | | 500 | 1067.26 (0.02) 0.00 | -0.11 (0.01) (0.01) 0.93 | -4.38 (0.01) (0.01) 0.82 |
| 0.2 | 3 | 1000, 100 | 522.85 (0.31) 0.00 | -0.21 (0.10) (0.09) 0.93 | -1.59 (0.10) (0.10) 0.89 |
| | | 500 | 523.28 (0.27) 0.00 | -0.06 (0.04) (0.04) 0.94 | -1.40 (0.04) (0.04) 0.82 |
| | | 5000, 100 | 523.12 (0.14) 0.00 | 0.01 (0.09) (0.09) 0.93 | -1.31 (0.10) (0.09) 0.89 |
| | | 500 | 523.16 (0.14) 0.00 | -0.01 (0.04) (0.04) 0.94 | -1.37 (0.04) (0.04) 0.83 |
| 0.2 | 0.2 | 1000, 100 | 565.11 (0.02) 0.00 | -0.23 (0.01) (0.01) 0.93 | -1.69 (0.01) (0.01) 0.90 |
| | | 500 | 565.54 (0.02) 0.00 | -0.08 (0.00) (0.00) 0.94 | -1.46 (0.00) (0.00) 0.86 |
| | | 5000, 100 | 565.48 (0.01) 0.00 | 0.03 (0.01) (0.01) 0.93 | -1.35 (0.01) (0.01) 0.89 |
| | | 500 | 565.46 (0.01) 0.00 | -0.03 (0.00) (0.00) 0.94 | -1.43 (0.00) (0.00) 0.86 |

Supplementary Table 5: Bias (%), empirical standard error (ESE), estimated sandwich standard error (SE), and coverage probability (CP) of 95% confidence interval for the interaction effect estimate $\widehat{\beta}_3$ based on 1000 simulations, under the EVS Scenario NI-NW in the true MEM. Working correlation specified as Unstructured in the GEE analysis for the outcome model.

| | | | Fitting Method | |
|---|---|---|---|---|
| | | Sample Size | Naive Method | Proposed Method |
| $\beta_1$ | $\beta_3$ | $n_1$, $n_2$ | Bias (%) (ESE) CP | Bias (%) (ESE) (SE) CP |
| 3 | 3 | 1000, 100 | -29.98 (0.12) 0.00 | 2.08 (0.46) (0.45) 0.94 |
| | | 500 | -30.15 (0.12) 0.00 | -0.03 (0.24) (0.24) 0.95 |
| | | 5000, 100 | -29.94 (0.05) 0.00 | 3.23 (0.48) (0.44) 0.95 |
| | | 500 | -30.04 (0.05) 0.00 | 0.27 (0.20) (0.19) 0.94 |
| 3 | 0.2 | 1000, 100 | -29.66 (0.02) 0.28 | 2.55 (0.04) (0.04) 0.96 |
| | | 500 | -30.60 (0.02) 0.25 | -0.67 (0.03) (0.03) 0.95 |
| | | 5000, 100 | -29.85 (0.01) 0.00 | 3.39 (0.03) (0.03) 0.95 |
| | | 500 | -30.04 (0.01) 0.00 | 0.27 (0.02) (0.02) 0.95 |
| 0.2 | 3 | 1000, 100 | -29.99 (0.10) 0.00 | 2.07 (0.46) (0.45) 0.94 |
| | | 500 | -30.12 (0.10) 0.00 | 0.005 (0.23) (0.23) 0.95 |
| | | 5000, 100 | -29.94 (0.05) 0.00 | 3.23 (0.48) (0.44) 0.95 |
| | | 500 | -30.04 (0.05) 0.00 | 0.27 (0.19) (0.19) 0.95 |
| 0.2 | 0.2 | 1000, 100 | -29.98 (0.01) 0.00 | 2.05 (0.03) (0.03) 0.95 |
| | | 500 | -30.25 (0.01) 0.00 | -0.19 (0.02) (0.02) 0.95 |
| | | 5000, 100 | -29.94 (0.00) 0.00 | 3.25 (0.03) (0.03) 0.95 |
| | | 500 | -30.01 (0.00) 0.00 | 0.31 (0.01) (0.01) 0.95 |

Supplementary Table 6: Bias (%), empirical standard error (ESE), estimated sandwich standard error (SE), and coverage probability (CP) of 95% confidence interval for the interaction effect estimate $\widehat{\beta}_3$ based on 1000 simulations, under the EVS Scenario IP-NW in the true MEM. Working correlation specified as Unstructured in the GEE analysis for the outcome model.

| | | | Fitting Method | | |
|---|---|---|---|---|---|
| | | Sample Size | Naive Method | Proposed, with Interaction | Proposed, no Interaction |
| $\beta_1$ | $\beta_3$ | $n_1$, $n_2$ | Bias (%) (ESE) CP | Bias (%) (ESE) (SE) CP | Bias (%) (ESE) (SE) CP |
| 3 | 3 | 1000, 100 | 546.85 (0.35) 0.00 | 0.17 (0.12) (0.11) 0.93 | 35.89 (1.75) (0.78) 0.07 |
| | | 500 | 546.60 (0.36) 0.00 | -0.04 (0.06) (0.06) 0.94 | 36.57 (1.70) (0.19) 0.04 |
| | | 5000, 100 | 546.50 (0.16) 0.00 | -0.11 (0.12) (0.11) 0.92 | 38.49 (2.18) (0.27) 0.03 |
| | | 500 | 546.67 (0.15) 0.00 | -0.01 (0.05) (0.05) 0.97 | 33.44 (1.74) (0.14) 0.01 |
| 3 | 0.2 | 1000, 100 | 1244.58 (2.55) 0.01 | 0.79 (0.02) (0.02) 0.93 | 171.19 (0.04) (0.03) 0.00 |
| | | 500 | 1200.22 (0.61) 0.02 | -0.10 (0.01) (0.01) 0.94 | 170.52 (0.02) (0.02) 0.00 |
| | | 5000, 100 | 1370.55 (7.35) 0.01 | 0.17 (0.02) (0.02) 0.92 | 170.74 (0.03) (0.03) 0.00 |
| | | 500 | 1138.93 (3.45) 0.01 | -0.01 (0.01) (0.01) 0.96 | 170.33 (0.02) (0.02) 0.00 |
| 0.2 | 3 | 1000, 100 | 506.98 (0.34) 0.00 | 0.13 (0.11) (0.10) 0.94 | 31.19 (0.78) (0.26) 0.07 |
| | | 500 | 506.71 (0.35) 0.00 | -0.04 (0.05) (0.05) 0.95 | 42.24 (12.72) (0.18) 0.02 |
| | | 5000, 100 | 506.57 (0.16) 0.00 | -0.12 (0.11) (0.10) 0.91 | 28.05 (0.29) (0.23) 0.02 |
| | | 500 | 506.77 (0.15) 0.00 | -0.01 (0.04) (0.05) 0.97 | 27.33 (0.14) (0.11) 0.00 |
| 0.2 | 0.2 | 1000, 100 | 593.47 (0.08) 0.00 | 0.15 (0.01) (0.01) 0.93 | 46.82 (0.02) (0.02) 0.00 |
| | | 500 | 590.30 (0.08) 0.00 | -0.06 (0.00) (0.00) 0.95 | 46.43 (0.01) (0.01) 0.00 |
| | | 5000, 100 | 606.79 (0.02) 0.00 | -0.09 (0.01) (0.01) 0.91 | 46.60 (0.02) (0.02) 0.00 |
| | | 500 | 606.87 (0.02) 0.00 | -0.02 (0.00) (0.00) 0.96 | 46.35 (0.01) (0.01) 0.00 |

Supplementary Table 7: Bias (%), empirical standard error (ESE), estimated sandwich standard error (SE), and coverage probability (CP) of 95% confidence interval for the interaction effect estimate $\widehat{\beta}_3$ based on 1000 simulations, under the EVS Scenario NI-NW in the true MEM. Working correlation specified as Independence in the GEE analysis for the outcome model.

| | | | Fitting Method | |
|---|---|---|---|---|
| | | Sample Size | Naive Method | Proposed Method |
| $\beta_1$ | $\beta_3$ | $n_1$, $n_2$ | Bias (%) (ESE) CP | Bias (%) (ESE) (SE) CP |
| 3 | 3 | 1000, 100 | -30.07 (0.15) 0.00 | 1.92 (0.48) (0.47) 0.94 |
| | | 500 | -30.29 (0.15) 0.00 | -0.24 (0.27) (0.28) 0.96 |
| | | 5000, 100 | -29.89 (0.07) 0.00 | 3.31 (0.48) (0.44) 0.95 |
| | | 500 | -29.99 (0.07) 0.00 | 0.34 (0.20) (0.20) 0.95 |
| 3 | 0.2 | 1000, 100 | -29.94 (0.03) 0.39 | 2.07 (0.05) (0.05) 0.95 |
| | | 500 | -31.01 (0.03) 0.36 | -1.29 (0.04) (0.04) 0.95 |
| | | 5000, 100 | -29.79 (0.01) 0.00 | 3.49 (0.04) (0.03) 0.95 |
| | | 500 | -29.90 (0.01) 0.00 | 0.46 (0.02) (0.02) 0.95 |
| 0.2 | 3 | 1000, 100 | -30.08 (0.14) 0.00 | 1.91 (0.47) (0.46) 0.94 |
| | | 500 | -30.24 (0.14) 0.00 | -0.18 (0.26) (0.26) 0.96 |
| | | 5000, 100 | -29.90 (0.06) 0.00 | 3.30 (0.48) (0.44) 0.95 |
| | | 500 | -30.00 (0.06) 0.00 | 0.33 (0.20) (0.20) 0.96 |
| 0.2 | 0.2 | 1000, 100 | -30.01 (0.01) 0.00 | 2.00 (0.03) (0.03) 0.95 |
| | | 500 | -30.32 (0.01) 0.00 | -0.30 (0.02) (0.02) 0.95 |
| | | 5000, 100 | -29.90 (0.01) 0.00 | 3.30 (0.03) (0.03) 0.95 |
| | | 500 | -30.00 (0.01) 0.00 | 0.32 (0.01) (0.01) 0.96 |

Supplementary Table 8: Bias (%), empirical standard error (ESE), estimated sandwich standard error (SE), and coverage probability (CP) of 95% confidence interval for the interaction effect estimate $\widehat{\beta}_3$ based on 1000 simulations, under the EVS Scenario IP-NW in the true MEM. Working correlation specified as Independence in the GEE analysis for the outcome model.

| | | | Fitting Method | | |
|---|---|---|---|---|---|
| | | Sample Size | Naive Method | Proposed, with Interaction | Proposed, no Interaction |
| $\beta_1$ | $\beta_3$ | $n_1$, $n_2$ | Bias (%) (ESE) CP | Bias (%) (ESE) (SE) CP | Bias (%) (ESE) (SE) CP |
| 3 | 3 | 1000, 100 | 588.92 (0.37) 0.00 | 0.15 (0.13) (0.13) 0.92 | 52.67 (0.31) (0.28) 0.00 |
| | | 500 | 588.70 (0.37) 0.00 | -0.09 (0.07) (0.07) 0.95 | 52.55 (0.15) (0.14) 0.00 |
| | | 5000, 100 | 588.43 (0.17) 0.00 | -0.10 (0.13) (0.12) 0.92 | 52.61 (0.28) (0.27) 0.00 |
| | | 500 | 588.67 (0.16) 0.00 | -0.05 (0.05) (0.05) 0.96 | 52.44 (0.13) (0.13) 0.00 |
| 3 | 0.2 | 1000, 100 | 1209.38 (0.04) 0.00 | 0.81 (0.03) (0.02) 0.93 | 174.04 (0.04) (0.03) 0.00 |
| | | 500 | 1209.05 (0.04) 0.00 | -0.17 (0.01) (0.01) 0.94 | 173.82 (0.02) (0.02) 0.00 |
| | | 5000, 100 | 1209.00 (0.02) 0.00 | 0.22 (0.03) (0.02) 0.93 | 173.99 (0.03) (0.03) 0.00 |
| | | 500 | 1209.09 (0.02) 0.00 | -0.08 (0.01) (0.01) 0.95 | 173.82 (0.02) (0.02) 0.00 |
| 0.2 | 3 | 1000, 100 | 547.57 (0.36) 0.00 | 0.11 (0.12) (0.11) 0.92 | 44.58 (0.29) (0.26) 0.00 |
| | | 500 | 547.34 (0.36) 0.00 | -0.08 (0.06) (0.06) 0.96 | 44.46 (0.14) (0.13) 0.00 |
| | | 5000, 100 | 547.05 (0.16) 0.00 | -0.12 (0.11) (0.11) 0.92 | 44.51 (0.26) (0.25) 0.00 |
| | | 500 | 547.30 (0.16) 0.00 | -0.05 (0.05) (0.05) 0.97 | 44.35 (0.12) (0.12) 0.00 |
| 0.2 | 0.2 | 1000, 100 | 589.06 (0.03) 0.00 | 0.17 (0.01) (0.01) 0.93 | 52.69 (0.02) (0.02) 0.00 |
| | | 500 | 588.62 (0.03) 0.00 | -0.10 (0.01) (0.01) 0.95 | 52.53 (0.01) (0.01) 0.00 |
| | | 5000, 100 | 588.41 (0.01) 0.00 | -0.09 (0.01) (0.01) 0.93 | 52.61 (0.02) (0.02) 0.00 |
| | | 500 | 588.67 (0.01) 0.00 | -0.05 (0.00) (0.00) 0.96 | 52.45 (0.01) (0.01) 0.00 |

Supplementary Table 9: Bias (%), empirical standard error (ESE), estimated sandwich standard error (SE), and coverage probability (CP) of 95% confidence interval for the interaction effect estimate $\widehat{\beta}_3$ based on 1000 simulations, under the IVS Scenario NI-NW in the true MEM. Working correlation specified as Unstructured in the GEE analysis for the outcome model.

| | | | Fitting Method | |
|---|---|---|---|---|
| | | Sample Size | Naive Method | Proposed Method |
| $\beta_1$ | $\beta_3$ | $n_1$, $n_2$ | Bias (%) (ESE) CP | Bias (%) (ESE) (SE) CP |
| 3 | 3 | 1000, 100 | -30.00 (0.11) 0.00 | 1.99 (0.46) (0.44) 0.94 |
| | | 500 | -29.82 (0.09) 0.00 | 0.56 (0.22) (0.21) 0.94 |
| | | 5000, 100 | -30.04 (0.05) 0.00 | 2.29 (0.45) (0.43) 0.93 |
| | | 500 | -30.08 (0.05) 0.00 | 0.21 (0.18) (0.19) 0.95 |
| 3 | 0.2 | 1000, 100 | -29.79 (0.02) 0.21 | 2.42 (0.04) (0.04) 0.95 |
| | | 500 | -29.40 (0.02) 0.12 | 1.17 (0.03) (0.03) 0.95 |
| | | 5000, 100 | -30.14 (0.01) 0.00 | 2.13 (0.03) (0.03) 0.94 |
| | | 500 | -30.33 (0.01) 0.00 | -0.17 (0.02) (0.02) 0.96 |
| 0.2 | 3 | 1000, 100 | -30.01 (0.10) 0.00 | 1.97 (0.45) (0.44) 0.94 |
| | | 500 | -29.85 (0.08) 0.00 | 0.52 (0.21) (0.20) 0.94 |
| | | 5000, 100 | -30.03 (0.05) 0.00 | 2.30 (0.45) (0.43) 0.94 |
| | | 500 | -30.06 (0.04) 0.00 | 0.23 (0.18) (0.19) 0.96 |
| 0.2 | 0.2 | 1000, 100 | -30.07 (0.01) 0.00 | 1.89 (0.03) (0.03) 0.95 |
| | | 500 | -29.79 (0.01) 0.00 | 0.59 (0.02) (0.02) 0.96 |
| | | 5000, 100 | -30.03 (0.00) 0.00 | 2.30 (0.03) (0.03) 0.94 |
| | | 500 | -30.16 (0.00) 0.00 | 0.10 (0.01) (0.01) 0.96 |

Supplementary Table 10: Bias (%), empirical standard error (ESE), estimated sandwich standard error (SE), and coverage probability (CP) of 95% confidence interval for the interaction effect estimate $\widehat{\beta}_3$ based on 1000 simulations, under the IVS Scenario IP-NW in the true MEM. Working correlation specified as Unstructured in the GEE analysis for the outcome model.

| | | | Fitting Method | | |
|---|---|---|---|---|---|
| | | Sample Size | Naive Method | Proposed, with Interaction | Proposed, no Interaction |
| $\beta_1$ | $\beta_3$ | $n_1$, $n_2$ | Bias (%) (ESE) CP | Bias (%) (ESE) (SE) CP | Bias (%) (ESE) (SE) CP |
| 3 | 3 | 1000, 100 | 546.43 (0.34) 0.00 | -0.18 (0.12) (0.11) 0.92 | 41.11 (2.55) (0.33) 0.05 |
| | | 500 | 546.85 (0.29) 0.00 | -0.02 (0.05) (0.05) 0.93 | 25.30 (6.69) (0.19) 0.03 |
| | | 5000, 100 | 546.94 (0.16) 0.00 | 0.05 (0.12) (0.11) 0.93 | 38.03 (1.73) (0.27) 0.02 |
| | | 500 | 546.80 (0.15) 0.00 | -0.04 (0.05) (0.05) 0.95 | 31.00 (0.62) (0.13) 0.02 |
| 3 | 0.2 | 1000, 100 | 1215.12 (2.18) 0.02 | -0.29 (0.02) (0.02) 0.93 | 170.77 (0.04) (0.03) 0.00 |
| | | 500 | 1195.45 (1.17) 0.01 | -0.07 (0.01) (0.01) 0.94 | 170.56 (0.02) (0.02) 0.00 |
| | | 5000, 100 | 1190.55 (0.77) 0.02 | 0.34 (0.02) (0.02) 0.92 | 171.73 (0.03) (0.03) 0.00 |
| | | 500 | 1035.41 (9.31) 0.00 | -0.11 (0.01) (0.01) 0.94 | 170.61 (0.02) (0.02) 0.00 |
| 0.2 | 3 | 1000, 100 | 506.57 (0.33) 0.00 | -0.17 (0.11) (0.10) 0.93 | 29.92 (0.51) (0.33) 0.05 |
| | | 500 | 506.97 (0.28) 0.00 | -0.01 (0.05) (0.05) 0.93 | 33.32 (3.64) (0.13) 0.01 |
| | | 5000, 100 | 507.02 (0.16) 0.00 | 0.03 (0.11) (0.10) 0.93 | 28.65 (0.26) (0.23) 0.02 |
| | | 500 | 506.89 (0.15) 0.00 | -0.03 (0.05) (0.05) 0.95 | 27.38 (0.15) (0.11) 0.00 |
| 0.2 | 0.2 | 1000, 100 | 593.62 (0.08) 0.00 | -0.14 (0.01) (0.01) 0.93 | 46.50 (0.02) (0.02) 0.00 |
| | | 500 | 599.92 (0.06) 0.00 | -0.00 (0.00) (0.00) 0.94 | 46.52 (0.01) (0.01) 0.00 |
| | | 5000, 100 | 607.60 (0.02) 0.00 | 0.05 (0.01) (0.01) 0.93 | 47.12 (0.02) (0.02) 0.00 |
| | | 500 | 607.29 (0.02) 0.00 | -0.04 (0.00) (0.00) 0.94 | 46.53 (0.01) (0.01) 0.00 |

Supplementary Table 11: Bias (%), empirical standard error (ESE), estimated sandwich standard error (SE), and coverage probability (CP) of 95% confidence interval for the interaction effect estimate $\widehat{\beta}_3$ based on 1000 simulations, under the IVS Scenario NI-NW in the true MEM. Working correlation specified as Independence in the GEE analysis for the outcome model.

| | | | Fitting Method | |
|---|---|---|---|---|
| | | Sample Size | Naive Method | Proposed Method |
| $\beta_1$ | $\beta_3$ | $n_1$, $n_2$ | Bias (%) (ESE) CP | Bias (%) (ESE) (SE) CP |
| 3 | 3 | 1000, 100 | -30.00 (0.15) 0.00 | 1.99 (0.48) (0.46) 0.95 |
| | | 500 | -29.81 (0.12) 0.00 | 0.58 (0.25) (0.24) 0.94 |
| | | 5000, 100 | -30.03 (0.07) 0.00 | 2.30 (0.46) (0.43) 0.94 |
| | | 500 | -30.13 (0.06) 0.00 | 0.14 (0.19) (0.20) 0.96 |
| 3 | 0.2 | 1000, 100 | -30.07 (0.03) 0.35 | 1.99 (0.05) (0.05) 0.94 |
| | | 500 | -29.41 (0.02) 0.23 | 1.17 (0.03) (0.03) 0.95 |
| | | 5000, 100 | -30.15 (0.01) 0.00 | 2.11 (0.03) (0.03) 0.95 |
| | | 500 | -30.44 (0.01) 0.00 | -0.32 (0.02) (0.02) 0.96 |
| 0.2 | 3 | 1000, 100 | -30.00 (0.13) 0.00 | 1.98 (0.47) (0.45) 0.96 |
| | | 500 | -29.83 (0.11) 0.00 | 0.54 (0.23) (0.23) 0.94 |
| | | 5000, 100 | -30.02 (0.06) 0.00 | 2.31 (0.45) (0.43) 0.94 |
| | | 500 | -30.11 (0.06) 0.00 | 0.16 (0.19) (0.19) 0.96 |
| 0.2 | 0.2 | 1000, 100 | -30.13 (0.01) 0.00 | 1.81 (0.03) (0.03) 0.95 |
| | | 500 | -29.81 (0.01) 0.00 | 0.57 (0.02) (0.02) 0.96 |
| | | 5000, 100 | -30.04 (0.01) 0.00 | 2.28 (0.03) (0.03) 0.94 |
| | | 500 | -30.17 (0.00) 0.00 | 0.08 (0.01) (0.01) 0.96 |

Supplementary Table 12: Bias (%), empirical standard error (ESE), estimated sandwich standard error (SE), and coverage probability (CP) of 95% confidence interval for the interaction effect estimate $\widehat{\beta}_3$ based on 1000 simulations, under the IVS Scenario IP-NW in the true MEM. Working correlation specified as Independence in the GEE analysis for the outcome model.

| | | | Fitting Method | | |
|---|---|---|---|---|---|
| | | Sample Size | Naive Method | Proposed, with Interaction | Proposed, no Interaction |
| $\beta_1$ | $\beta_3$ | $n_1$, $n_2$ | Bias (%) (ESE) CP | Bias (%) (ESE) (SE) CP | Bias (%) (ESE) (SE) CP |
| 3 | 3 | 1000, 100 | 588.54 (0.34) 0.00 | -0.18 (0.13) (0.12) 0.93 | 52.47 (0.29) (0.27) 0.00 |
| | | 500 | 588.82 (0.29) 0.00 | -0.01 (0.06) (0.06) 0.94 | 52.61 (0.13) (0.12) 0.00 |
| | | 5000, 100 | 588.96 (0.17) 0.00 | 0.06 (0.13) (0.12) 0.93 | 53.15 (0.28) (0.27) 0.00 |
| | | 500 | 588.73 (0.16) 0.00 | -0.06 (0.06) (0.06) 0.95 | 52.64 (0.13) (0.13) 0.00 |
| 3 | 0.2 | 1000, 100 | 1208.74 (0.04) 0.00 | -0.33 (0.03) (0.02) 0.92 | 173.61 (0.04) (0.03) 0.00 |
| | | 500 | 1208.98 (0.03) 0.00 | -0.06 (0.01) (0.01) 0.94 | 173.93 (0.02) (0.02) 0.00 |
| | | 5000, 100 | 1209.50 (0.02) 0.00 | 0.38 (0.02) (0.02) 0.92 | 175.03 (0.03) (0.03) 0.00 |
| | | 500 | 1209.18 (0.02) 0.00 | -0.16 (0.01) (0.01) 0.95 | 174.11 (0.02) (0.02) 0.00 |
| 0.2 | 3 | 1000, 100 | 547.20 (0.34) 0.00 | -0.17 (0.11) (0.11) 0.93 | 44.40 (0.27) (0.25) 0.00 |
| | | 500 | 547.47 (0.29) 0.00 | -0.01 (0.05) (0.05) 0.94 | 44.52 (0.12) (0.11) 0.00 |
| | | 5000, 100 | 547.59 (0.16) 0.00 | 0.04 (0.11) (0.11) 0.93 | 45.03 (0.27) (0.25) 0.00 |
| | | 500 | 547.37 (0.15) 0.00 | -0.05 (0.05) (0.05) 0.95 | 44.54 (0.12) (0.12) 0.00 |
| 0.2 | 0.2 | 1000, 100 | 588.61 (0.02) 0.00 | -0.16 (0.01) (0.01) 0.93 | 52.48 (0.02) (0.02) 0.00 |
| | | 500 | 588.76 (0.02) 0.00 | -0.02 (0.00) (0.00) 0.94 | 52.60 (0.01) (0.01) 0.00 |
| | | 5000, 100 | 588.94 (0.01) 0.00 | 0.07 (0.01) (0.01) 0.93 | 53.16 (0.02) (0.02) 0.00 |
| | | 500 | 588.74 (0.01) 0.00 | -0.05 (0.00) (0.00) 0.95 | 52.64 (0.01) (0.01) 0.00 |

# Web Appendix C Validation and Main Study Data Analysis Results

Supplementary Table 13: Basic characteristics of the validation study (108 participants, 195 person-months).

| Characteristic | Mean (SD) (min,max) |
|---|---|
| Monthly personal $PM_{2.5}$ of ambient origin ($\mu g/m^3$) | 10.5 (4.5) (3.3, 22.8) |
| Monthly spatio-temporal predicted $PM_{2.5}$ ($\mu g/m^3$) | 16.5 (4.3) (8.7, 24.4) |
| Age (years) | 67.5 (12.5) (30.0, 90.3) |
| | No. (%) |
| Number of records per person | |
| 1 | 65 (60) |
| $> 1$ | 43 (40) |

Supplementary Table 14: Basic characteristics of the NHS Cognitive Cohort (n=19002 nurses, N=47468 person-months).

| Characteristic | Mean (SD) | (min, max) |
|---|---|---|
| Averaged monthly spatio-temporal predicted $PM_{2.5}$ ($\mu g/m^3$) | 14.16 (3.02) | (1.86, 25.45) |
| Global cognitive score | 0.01 (0.66) | (-4.79, 2.56) |
| Baseline age (years) | 74.2 (2.28) | (70.1, 81.0) |
| | No. (%) | |
| Number of cognitive assessment per person | | |
| 1 | 2839 (15) | |
| 2 | 3860 (20) | |
| 3 | 12303 (65) | |
| Education | | |
| Registered nurse | 14773 (78) | |
| Bachelor of arts degree | 3142 (16) | |
| $\geq$ Master of arts degree | 1087 (6) | |
| Husband's education | | |
| $\leq$ High school | 7377 (39) | |
| College | 4107 (22) | |
| Graduate school | 2991 (16) | |
| Long-term averaged alcohol consumption (g/d) | | |
| 0 | 5663 (30) | |
| $0 - 5$ | 7229 (38) | |
| $5 - 15$ | 3518 (19) | |
| $> 15$ | 1888 (10) | |
| Long-term averaged level of physical activity (MET-hrs/wk) by quartile | | |
| Lowest | 4679 (25) | |
| Second | 4686 (25) | |
| Third | 4706 (25) | |
| Highest | 4679 (25) | |

Supplementary Table 15: Five-number summary of important characteristics of the NHS Cognitive Cohort and the validation study.

| Age (years) | | | | | |
|---|---|---|---|---|---|
| | Min | 1st Quartile | Median | 3rd Quartile | Max |
| MS | 70.10 | 72.33 | 74.00 | 75.92 | 81.00 |
| EVS | 30.00 | 60.80 | 70.00 | 75.00 | 90.30 |
| Monthly Spatio-Temporal Predicted $PM_{2.5}$ ($\mu g/m^3$) | | | | | |
| | Min | 1st Quartile | Median | 3rd Quartile | Max |
| MS | 1.86 | 12.02 | 14.11 | 16.44 | 25.45 |
| EVS | 8.69 | 13.32 | 16.65 | 19.60 | 24.37 |

Supplementary Table 16: Comparison of covariates between people with all cognitive assessments and those missing some cognitive assessments in the NHS Cognitive Cohort. Full and Missing refer to people who have/ do not have three cognitive assessments, respectively.

| Characteristic | Full<br>12303 nurses | Missing<br>6699 nurses |
|---|---|---|
| | Mean (SD) | Mean (SD) |
| Monthly Spatio-Temporal Predicted $PM_{2.5}$ ($\mu$g/m$^3$) | 14.15 (2.99) | 14.22 (3.13) |
| | No. (%) | No. (%) |
| Education | | |
| Registered nurse | 9522 (77) | 5251 (78) |
| Bachelor of arts degree | 2052 (17) | 1090 (16) |
| $\geq$ Master of arts degree | 729 (6) | 358 (6) |
| Husband's education | | |
| $\leq$ High school | 4903 (40) | 2474 (37) |
| College | 2741 (22) | 1366 (20) |
| Graduate school | 1928 (16) | 1063 (16) |
| Long-term average alcohol consumption (g/d) | | |
| 0 | 3537 (29) | 2126 (32) |
| $0 - 5$ | 4854 (39) | 2375 (35) |
| $5 - 15$ | 2336 (19) | 1182 (18) |
| $> 15$ | 1238 (10) | 650 (10) |
| Long-term average level of physical activity (MET-hrs/wk) by quartile | | |
| Lowest | 2917 (24) | 1762 (26) |
| Second | 3073 (25) | 1613 (24) |
| Third | 3131 (25) | 1575 (24) |
| Highest | 3068 (25) | 1611 (24) |

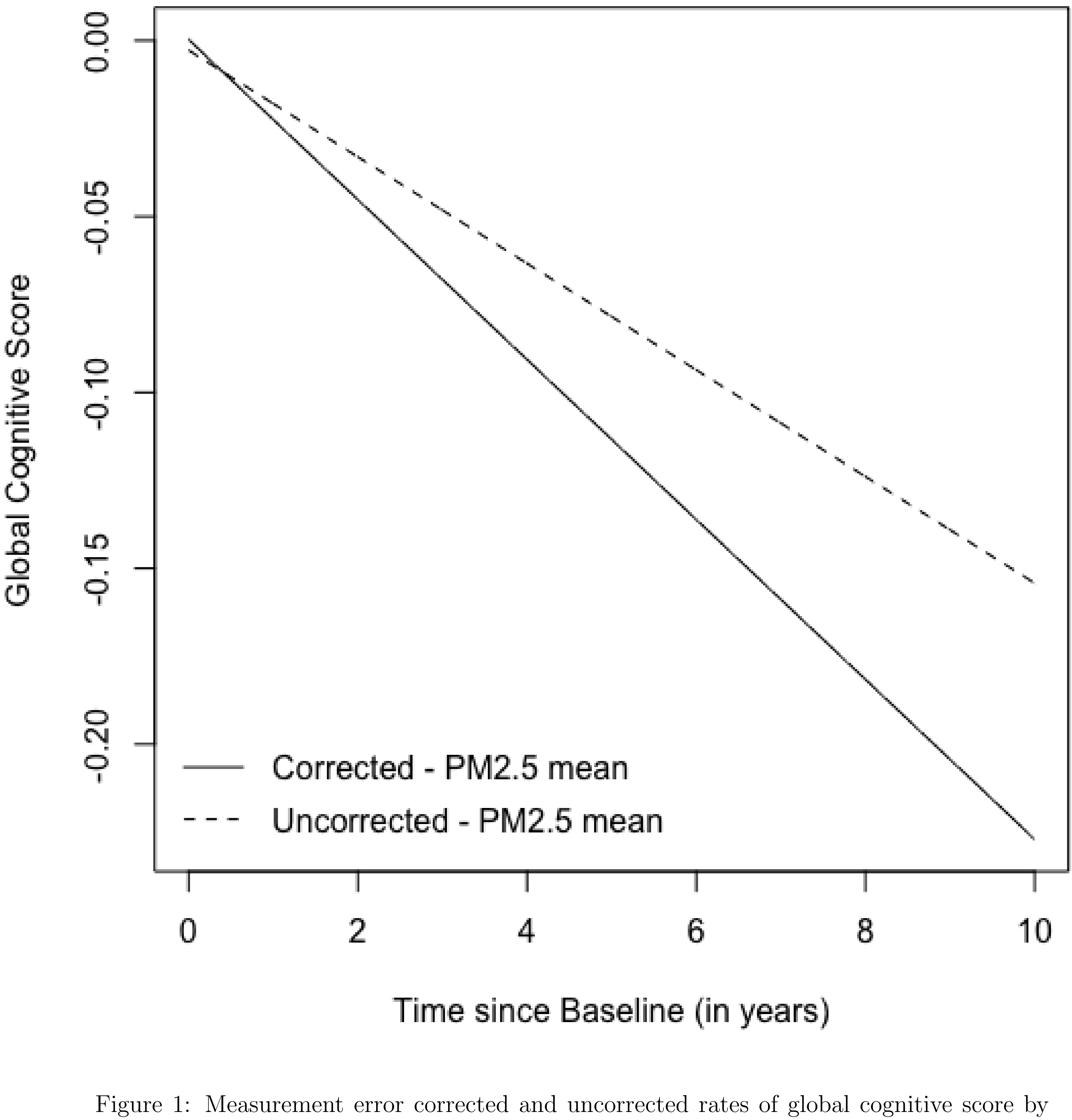


Figure 1: Measurement error corrected and uncorrected rates of global cognitive score by time since baseline. The longest follow-up time in the main study was 10 years. $PM_{2.5}$ mean was the mean (14 $\mu g/m^3$) level in the NHS Cognitive Cohort, and baseline age was set as the mean age (74.2 years) at baseline in the NHS Cognitive Cohort.

# References

Carroll, R. J., Ruppert, D., Stefanski, L. A., & Crainiceanu, C. M. (2006). *Measurement error in nonlinear models: A modern perspective*. Chapman; Hall/CRC.
